# Impact of nitroglycerin on 28-day mortality in ischemic stroke patients: a retrospective cohort study using the MIMIC-IV database

**DOI:** 10.3389/fneur.2025.1577700

**Published:** 2025-09-12

**Authors:** Huan Zuo, Xin Zuo, Linjing Wang

**Affiliations:** ^1^Department of Acupuncture, Heilongjiang University of Chinese Medicine, Harbin, China; ^2^Department of Acupuncture, The Second Affiliated Hospital of Heilongjiang University of Chinese Medicine, Harbin, China

**Keywords:** nitroglycerin, ischemic stroke, mortality, MIMIC-IV database, therapeutic potential

## Abstract

**Background:**

The impact of nitroglycerin (NTG) on short-term outcomes in ischemic stroke patients remains unclear. This study aimed to evaluate the association between NTG use—including route, timing, and duration—and 28-day in-hospital mortality.

**Methods:**

We conducted a retrospective cohort study of 3,434 ischemic stroke patients, including 356 who received NTG. Propensity score matching (1:1) was used to reduce confounding. Cox proportional hazards models, Kaplan–Meier analysis, and stratified analyses assessed the association between NTG use and mortality.

**Results:**

NTG use was associated with reduced 28-day mortality before (HR = 0.52, *p* < 0.001) and after matching (HR = 0.50, *p* = 0.003). Kaplan–Meier curves confirmed this benefit. Among administration routes, only intravenous drip (IV) was significantly associated with reduced mortality (*p* < 0.001). Early initiation within 24 h (*p* < 0.001) and a treatment duration of 1–3 days (*p* < 0.001) were also significantly associated with lower mortality. No benefit was observed for other routes, delayed initiation, or longer durations. Dose-stratified analysis showed no difference between low and high doses (*p* = 0.59).

**Conclusion:**

Intravenous drip NTG, started within 24 h and continued for 1–3 days, was significantly associated with lower 28-day mortality in ischemic stroke patients. These findings suggest a potential therapeutic window and warrant further prospective validation.

## 1 Introduction

Ischemic stroke, is a major contributor to global morbidity and mortality. Epidemiological studies indicate a high incidence and prevalence of ischemic stroke, especially in older adults and those with cardiovascular risk factors such as hypertension, diabetes, and atherosclerosis. The impact of ischemic stroke extends beyond physical impairment, frequently affecting cognitive function, emotional health, and overall quality of life. The burden on healthcare systems is substantial, driven by prolonged rehabilitation requirements and significant socioeconomic costs. Given this impact, ongoing research into preventive and therapeutic strategies remains essential (1–4).

Nitroglycerin (NTG), a nitrate compound synthesized in the 19th century, has since become essential for the treatment of angina pectoris and acute heart failure (5). Nitroglycerin acts as a vasodilator by releasing nitric oxide (NO), which relaxes smooth muscle and promotes both peripheral and coronary vasodilation. Clinically, it is used to relieve angina, manage hypertensive emergencies, and stabilize acute heart failure by enhancing myocardial oxygen delivery and reducing preload (6).

In addition to cardiovascular indications, nitroglycerin has been investigated for several off-label applications, including the reduction of vascular spasms, promotion of wound healing, and potential modulation of cerebral blood flow (7–9).

The NTG has several clinical applications as a nitric oxide donor and vasodilator in the management of ischemic stroke. It can be used for acute-phase blood pressure control, particularly within 2–6 h of ischemic stroke onset, via transdermal patch administration to lower blood pressure, potentially improve functional outcomes, and benefit patients who are unable to receive oral or intravenous medications (10). In a subgroup analysis of the ENOS trial, NTG was found to be not only safe but also associated with a significantly improved 90-day prognosis in ischemic stroke patients with severe carotid artery stenosis (≥70%) (mRS improvement; OR 0.56, 95% CI 0.34–0.93), suggesting its neuroprotective potential in this specific patient population (11). Furthermore, animal studies have demonstrated that intra-arterial injection of NTG in a middle cerebral artery occlusion (MCAo) model significantly reduces infarct volume, inhibits astrocyte activation, promotes neuronal survival, and improves motor function on postoperative day 1. These effects may be mediated not only by blood pressure reduction but also by nitric oxide–induced local neuroprotection (12). Collectively, these three studies underscore the potential clinical benefits of NTG in ischemic stroke, mediated through blood pressure reduction, enhanced collateral circulation, and direct neuroprotective effects across diverse patient populations and therapeutic time windows.

However, the role of nitroglycerin in ischemic stroke remains uncertain due to the distinct hemodynamic characteristics of cerebral perfusion compared to systemic circulation. While some studies suggest that nitroglycerin increases cerebral blood flow by reducing vascular resistance, others caution that it may induce hypotension, thereby decreasing mean cerebral perfusion (13). The impact of nitroglycerin on ischemic stroke outcomes remains poorly defined. Although nitroglycerin may influence recovery or complications through its systemic effects, existing research remains limited. This study aims to evaluate the potential effect of nitroglycerin on ischemic stroke prognosis using data from the MIMIC-IV database, thereby addressing a critical knowledge gap and informing future therapeutic strategies. As a publicly available resource, the MIMIC-IV database provides real-world clinical data that complement randomized controlled trials and offer broader evidence to support clinical decision-making.

## 2 Materials and methods

### 2.1 Data source

The Medical Information Mart for Intensive Care IV (MIMIC-IV) is a publicly available, de-identified clinical database that contains comprehensive patient data from the intensive care units (ICUs) at Beth Israel Deaconess Medical Center (14). The study cohort consists of patients admitted to the ICU between 2008 and 2022. The MIMIC-IV database includes a diverse adult population and provides detailed, real-time clinical data—such as vital signs, laboratory results, medications, procedures, and patient outcomes—facilitating in-depth analyses in critical care settings. To protect patient privacy, the database has been rigorously de-identified while preserving the level of data granularity necessary for research. This study adhered to all applicable ethical guidelines and regulatory requirements. Huan Zuo, a co-author of this study, completed all required training and certification exams to access the MIMIC-IV database [Record ID: 65378168 (for HZ)] and was responsible for data extraction, ensuring both ethical integrity and data reliability for subsequent analysis. All study procedures were conducted in accordance with relevant guidelines and regulations, including the ethical standards of the MIMIC-IV database and applicable institutional protocols.

### 2.2 Study population

**Figure 1:**
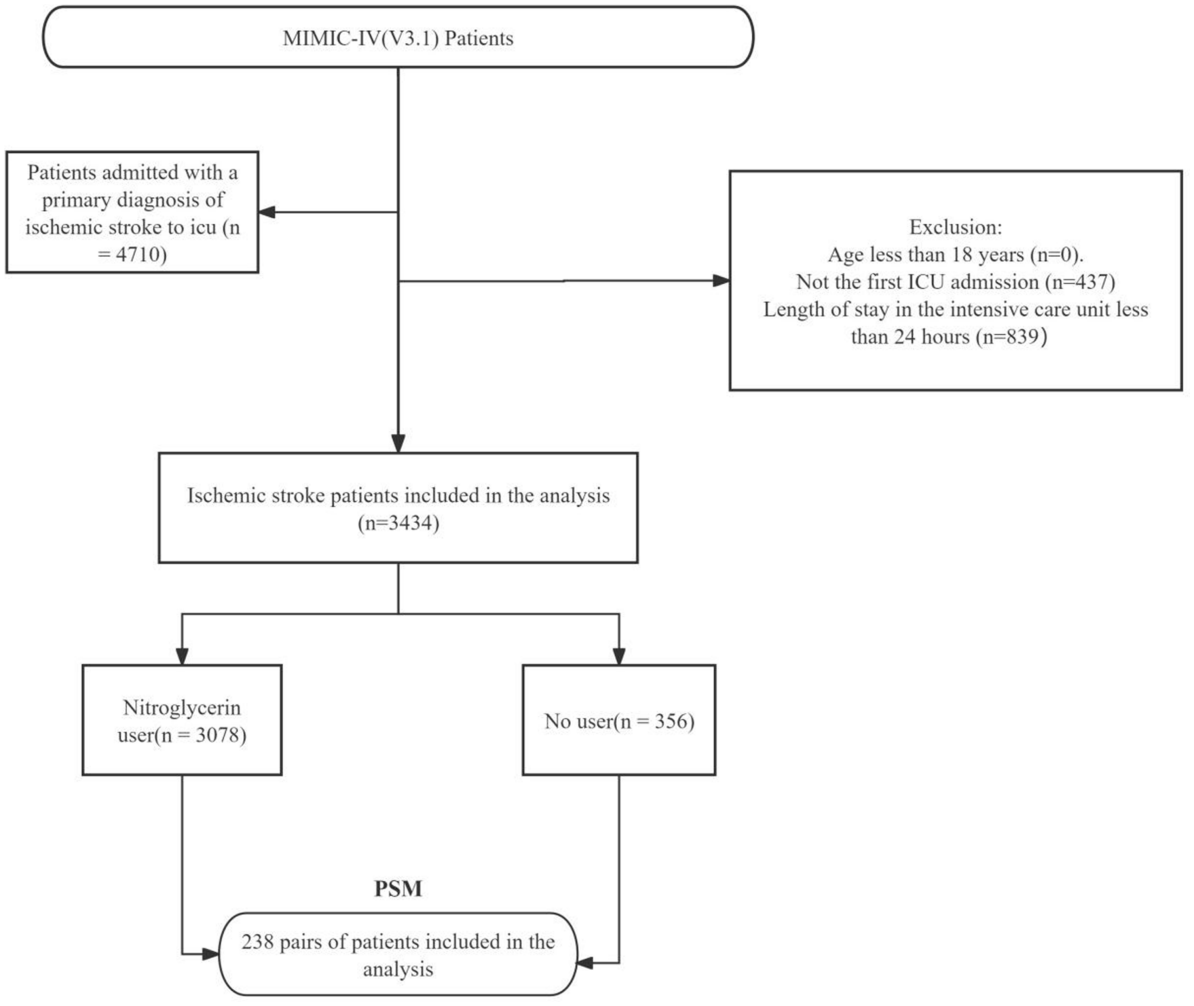
Flow-diagram illustrating patient inclusion in the study.

This study investigated the effect of nitroglycerin on the prognosis of patients with ischemic stroke, and the patient selection process was based on data from the MIMIC-IV(v3.1) database. Initially, 4,710 patients with a primary diagnosis of ischemic stroke were identified (Figure 1). The following exclusion criteria were applied: (1) patients under 18 years old (none excluded); (2) only the first ICU admission was included, resulting in the exclusion of 437 patients with multiple ICU admissions; and (3) patients with an ICU stay of less than 24 h were excluded (*n* = 839). After applying these criteria, 3,434 patients were eligible for analysis. Propensity score matching (PSM) was then performed, resulting in 238 matched patient pairs. This selection process ensured cohort homogeneity and enhanced the reliability of subsequent outcome analyses. To minimize potential bias due to missing data, variables with more than 15% missingness were excluded, while those with less than 15% were imputed using the random forest algorithm in the *missForest* R package (15–17). This approach improved group comparability and strengthened the validity of the analytical results.

### 2.3 Data collection

In this study, patient data were extracted from the MIMIC-IV database using PostgreSQL. To ensure consistency and comparability, only data from the first 24 h of hospital admission were collected, reflecting the patients’ initial clinical condition.

Collected variables included demographic information (age, gender, race), vital signs (heart rate, systolic and diastolic blood pressure, mean arterial pressure, respiratory rate, temperature, oxygen saturation), laboratory tests (glucose, BUN, potassium, sodium, creatinine, WBC, RBC, hemoglobin, hematocrit, platelet count, MCV, MCH, MCHC, RDW, chloride, anion gap, bicarbonate, calcium, PT, PTT, INR), clinical scores (SOFA, SAPSII, GCS, Charlson Comorbidity Index), comorbidities (myocardial infarction, renal disease, liver disease, congestive heart failure, peripheral vascular disease, dementia, chronic pulmonary disease, rheumatic disease, peptic ulcer disease, diabetes, hyperlipidemia, atrial fibrillation, hypertension, acute kidney injury), and treatments (use of ventilator, CRRT). All variables were retrieved using structured queries and represent the earliest available values within the first 24 h after ICU admission.

Information on nitroglycerin administration was extracted from the MIMIC-IV database. Specifically, data were collected on the route of administration (e.g., intravenous drip [IV], sublingual [SL], transdermal/oral/tube feeding [TD/PO/TP]), the timing of initial administration (i.e., within 1 day, 1–3 days, or ≥4 days after hospital admission), and the duration of use (i.e., <1 day, 1–3 days, or ≥4 days). These variables were derived by linking medication administration records to admission timestamps, allowing classification of each patient’s nitroglycerin exposure characteristics for further analysis.

### 2.4 Outcomes

The primary outcome of the study was 28-day mortality, defined as patient survival within 28 days following hospital admission.

### 2.5 Nitroglycerin exposure

Nitroglycerin exposure was defined as the first total dose of nitroglycerin medication administered after admission to the intensive care unit.

### 2.6 Statistical analysis

All statistical analyses were conducted using R software. Continuous variables were reported as mean ± standard deviation (SD) or median with interquartile range (IQR), and compared using Student’s t-test or the Mann–Whitney U test, as appropriate. Categorical variables were summarized as counts (percentages) and compared using the chi-square test or Fisher’s exact test. Propensity score matching (PSM) was performed using 1:1 nearest-neighbor matching with a caliper width of 0.1 to minimize confounding. Covariate balance before and after matching was assessed using standardized mean differences (SMDs), with values <0.1 considered indicative of adequate balance.

Univariable Cox proportional hazards regression was used to identify risk factors for 28-day mortality, and variables with a *p* < 0.05 were included in the multivariable Cox regression models. Three models were constructed: Model 1 (unadjusted); Model 2 (adjusted for age, sex, and race); and Model 3 (additionally adjusted for significant covariates identified in univariable analysis). Kaplan–Meier survival curves and log-rank tests were used to compare survival between groups. Receptor operating characteristic (ROC) analysis was used to determine the optimal threshold for the cumulative dose of nitroglycerin, and groups were grouped according to this threshold.

Subgroup analyses were conducted to assess the consistency of the association between nitroglycerin use and 28-day mortality across predefined subgroups. Interaction terms were included to test for effect modification. A two-sided *p* value < 0.05 was considered statistically significant.

## 3 Results

### 3.1 Patient characteristics

A total of 3,434 patients were included in this study. Of these, 3,078 did not receive nitroglycerin, while 356 received the treatment. Routes of nitroglycerin administration were as follows: intravenous drip (IV DRIP) in 320 patients (89.8%), sublingual (SL) in 26 (7.3%), transcutaneous (TP) in 8 (2.2%), transdermal patch (TD) in 1 (0.1%), and oral (PO) in 1 (0.1%) (Supplementary Table S1). Supplementary Table S1 further summarizes the timing of nitroglycerin initiation relative to ICU admission, as well as the total duration of use within 28 days. The optimal cutoff value for cumulative intravenous nitroglycerin dose (100.3 mg) was identified using receiver operating characteristic (ROC) curve analysis. Accordingly, 145 patients received a dose ≤100.3 mg, while 175 received >100.3 mg.

**Figure 2:**
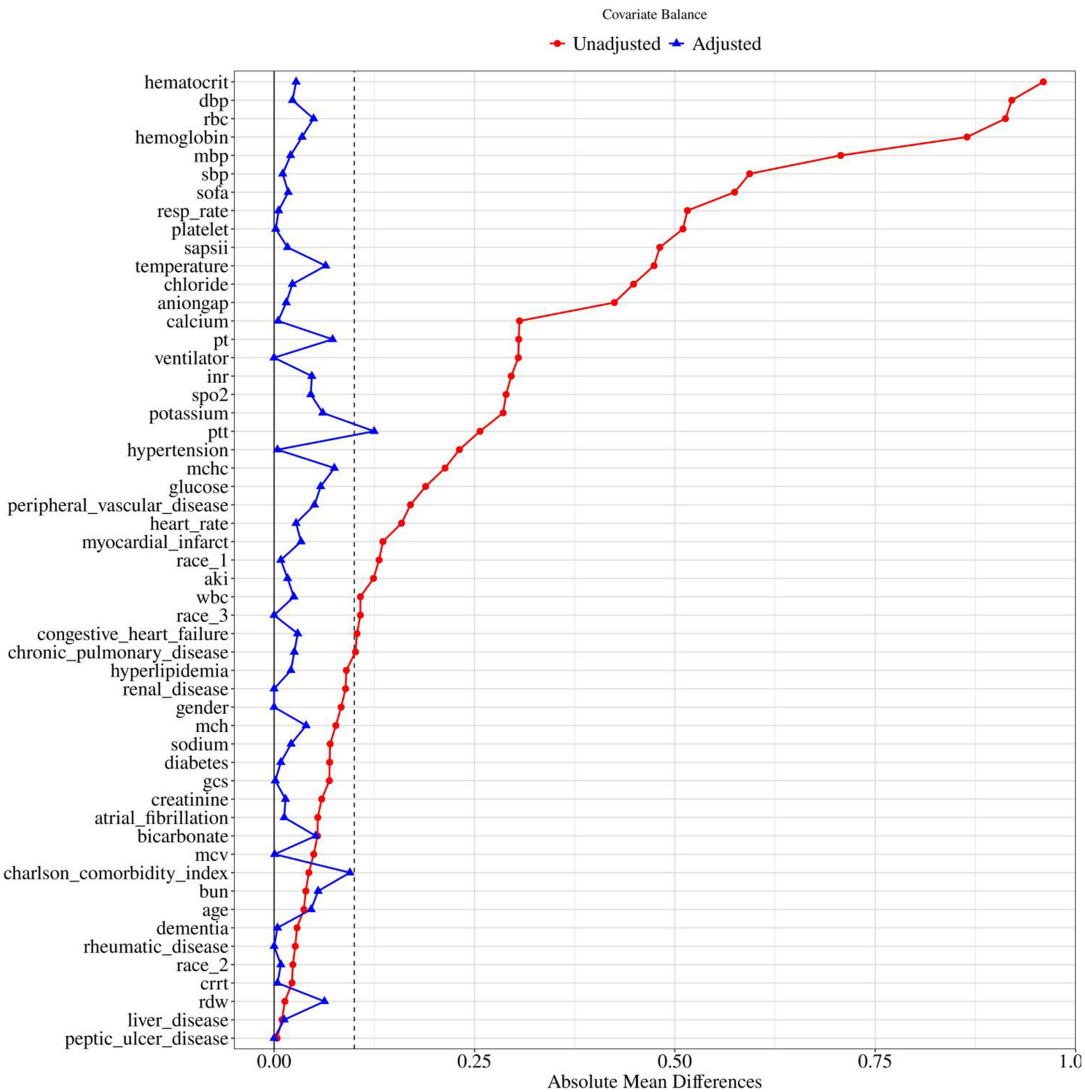
Standardized mean difference of pre and post PSM variables for nitroglycerin use. BUN, blood urea nitrogen; RDW, red cell distribution width; WBC, white blood cell count; MCHC, mean corpuscular hemoglobin concentration; RDW, red cell distribution width; RBC, red blood cells; MCV, mean corpuscular volume; MCH, mean corpuscular hemoglobin; INR, international normalized ratio; PT, prothrombin time; PTT, partial thromboplastin time; SBP, systolic blood pressure; DBP, diastolic blood pressure; MBP, mean blood pressure; Resp rate, respiratory rate; SpO₂, peripheral oxygen saturation; AKI, acute kidney injury; CRRT, continuous renal replacement therapy; SOFA, sequential organ failure assessment; GCS, Glasgow Coma Scale; SAPSII, simplified acute physiology score II.

To control for confounding, propensity score matching (PSM) was performed using 1:1 nearest-neighbor matching with a caliper width of 0.1. Before matching, most covariates were significantly imbalanced between groups, with several standardized mean differences (SMDs) > 0.1, indicating substantial confounding. After matching, the SMDs of all covariates were substantially reduced, with most falling below 0.1, indicating that PSM effectively balanced baseline characteristics between the two groups (Table 1 and Figure 2). This supports the validity of subsequent comparisons, as observed differences are more likely attributable to the intervention rather than residual confounding.

**Table 1:** Baseline characteristics of Ischemic Stroke patients before and after PSM.

Variables	Before PSM					After PSM				
	Total (*n* = 3,434)	No nitroglycerin (*n* = 3,078)	Use nitroglycerin (*n* = 356)	*P*	SMD	Total (*n* = 476)	No Nitroglycerin (*n* = 238)	Use nitroglycerin (n = 238)	*P*	SMD
Age	71.00 (61.00, 82.00)	71.00 (60.00, 82.00)	72.00 (63.00, 80.00)	0.921	0.037	73.00 (63.00, 81.00)	73.00 (62.25, 82.00)	72.00 (63.00, 80.00)	0.501	−0.045
Gender, *n* (%)				0.003					1.000	
Female	1,685 (49.07)	1,537 (49.94)	148 (41.57)		−0.170	212 (44.54)	106 (44.54)	106 (44.54)		0.000
Man	1749 (50.93)	1,541 (50.06)	208 (58.43)		0.170	264 (55.46)	132 (55.46)	132 (55.46)		0.000
Race, *n* (%)				<0.001					0.943	
White	2,153 (62.7)	1888 (61.34)	265 (74.44)		0.300	336 (70.59)	167 (70.17)	169 (71.01)		0.019
Black	342 (9.96)	314 (10.20)	28 (7.87)		−0.087	38 (7.98)	20 (8.40)	18 (7.56)		−0.032
Others	939 (27.34)	876 (28.46)	63 (17.70)		−0.282	102 (21.43)	51 (21.43)	51 (21.43)		0.000
Vital signs										
Heart Rate, bpm	80.00 (69.00, 93.00)	80.00 (69.00, 94.00)	80.00 (70.00, 88.00)	0.151	−0.159	79.00 (68.00, 91.00)	78.00 (66.25, 93.00)	80.00 (70.00, 89.00)	0.608	−0.025
SBP, mmHg	139.00 (122.00, 155.00)	141.00 (124.00, 156.00)	121.00 (106.75, 139.25)	<0.001	−0.593	130.00 (111.00, 149.00)	130.00 (114.00, 148.00)	130.00 (110.00, 150.00)	0.717	0.010
DBP, mmHg	75.00 (64.00, 89.00)	77.00 (66.00, 90.00)	60.50 (52.75, 70.25)	<0.001	−0.920	65.00 (54.75, 76.00)	66.00 (55.00, 78.00)	64.00 (54.00, 75.00)	0.412	−0.021
MBP, mmHg	93.00 (81.00, 106.00)	95.00 (83.00, 107.00)	81.00 (71.00, 92.00)	<0.001	−0.707	86.00 (73.75, 96.00)	86.00 (73.25, 96.00)	85.50 (74.00, 95.00)	0.973	0.019
Resp Rate, bpm	18.00 (15.00, 21.00)	18.00 (15.00, 22.00)	16.00 (14.00, 18.00)	<0.001	−0.516	17.00 (14.00, 20.00)	17.00 (14.00, 20.00)	16.00 (14.00, 20.00)	0.757	−0.005
Temperature, °C	36.72 (36.50, 37.00)	36.78 (36.50, 37.02)	36.50 (36.00, 36.83)	<0.001	−0.474	36.67 (36.32, 36.94)	36.72 (36.39, 36.98)	36.56 (36.23, 36.89)	0.030	−0.066
SpO2,%	98.00 (96.00, 100.00)	98.00 (96.00, 100.00)	100.00 (97.00, 100.00)	<0.001	0.289	99.00 (96.00, 100.00)	98.00 (96.00, 100.00)	99.00 (96.00, 100.00)	0.284	0.040
Laboratory index										
Glucose, mg/dL	125.00 (103.00, 160.00)	124.00 (102.00, 159.00)	135.50 (111.75, 173.00)	<0.001	0.189	134.00 (110.00, 178.25)	133.50 (110.00, 178.75)	134.50 (110.00, 178.00)	0.802	−0.054
BUN, mg/dL	17.00 (12.00, 24.00)	16.50 (12.00, 23.00)	18.00 (13.00, 25.00)	0.009	0.040	19.00 (14.00, 26.00)	19.00 (14.00, 26.75)	19.00 (14.00, 26.00)	0.697	0.050
Potassium, mEq/L	4.00 (3.70, 4.40)	4.00 (3.70, 4.40)	4.20 (3.90, 4.60)	<0.001	0.286	4.10 (3.80, 4.50)	4.10 (3.80, 4.50)	4.20 (3.90, 4.50)	0.207	0.060
Sodium, mEq/L	139.00 (137.00, 142.00)	139.00 (137.00, 142.00)	139.00 (136.00, 141.00)	0.263	−0.070	139.00 (136.00, 141.00)	139.00 (136.00, 142.00)	139.00 (136.00, 141.00)	0.910	0.019
Creatinine, mEq/L	0.90 (0.70, 1.20)	0.90 (0.70, 1.20)	0.90 (0.80, 1.20)	0.094	0.059	1.00 (0.80, 1.33)	1.00 (0.80, 1.40)	0.90 (0.80, 1.30)	0.241	−0.013
WBC, K/uL	9.70 (7.50, 12.90)	9.70 (7.50, 12.70)	10.25 (7.40, 14.30)	0.037	0.108	10.00 (7.20, 13.85)	9.90 (7.00, 13.38)	10.05 (7.30, 14.25)	0.636	0.024
MCHC, %	33.00 (32.00, 33.90)	32.90 (32.00, 33.90)	33.20 (32.18, 34.30)	<0.001	0.213	33.10 (32.00, 34.00)	33.10 (32.00, 34.00)	33.15 (32.00, 34.00)	0.609	0.073
RDW, %	13.80 (13.10, 14.80)	13.80 (13.10, 14.80)	13.90 (13.20, 14.80)	0.377	−0.013	14.00 (13.20, 15.00)	14.10 (13.40, 15.10)	13.90 (13.20, 15.00)	0.160	−0.057
RBC, M/uL	4.02 (3.49, 4.49)	4.08 (3.60, 4.53)	3.31 (2.83, 3.93)	<0.001	−0.913	3.60 (3.09, 4.11)	3.63 (3.16, 4.09)	3.58 (3.06, 4.12)	0.449	−0.050
Platelet, K/uL	209.00 (164.00, 266.00)	214.00 (169.00, 270.00)	169.00 (128.75, 219.25)	<0.001	−0.510	189.00 (145.00, 242.25)	194.00 (143.25, 240.50)	184.50 (146.00, 247.50)	0.686	0.002
MCV, fL	91.00 (87.00, 95.00)	91.00 (87.00, 95.00)	91.00 (86.00, 94.00)	0.323	−0.049	91.00 (86.00, 94.00)	91.00 (86.25, 94.75)	91.00 (86.00, 94.00)	0.686	−0.001
MCH, pg	30.10 (28.60, 31.40)	30.10 (28.60, 31.40)	30.10 (28.80, 31.60)	0.404	0.077	30.05 (28.60, 31.50)	30.10 (28.60, 31.37)	30.00 (28.63, 31.58)	0.804	0.039
Hematocrit, %	12.00 (10.30, 13.50)	12.20 (10.60, 13.60)	9.80 (8.50, 11.70)	<0.001	−0.865	10.60 (9.10, 12.50)	10.75 (9.33, 12.50)	10.40 (9.00, 12.30)	0.370	−0.034
Hemoglobin, g/dL	36.40 (31.70, 40.50)	37.10 (32.70, 40.80)	29.85 (25.90, 34.82)	<0.001	−0.960	31.70 (28.08, 37.50)	32.70 (28.47, 37.50)	31.20 (28.00, 37.40)	0.454	−0.028
INR	1.20 (1.10, 1.30)	1.20 (1.10, 1.30)	1.30 (1.10, 1.50)	<0.001	0.296	1.20 (1.10, 1.40)	1.20 (1.10, 1.40)	1.20 (1.10, 1.40)	0.631	−0.049
PT	12.80 (11.80, 14.38)	12.60 (11.70, 14.10)	14.20 (12.60, 16.60)	<0.001	0.305	13.30 (12.30, 15.50)	13.30 (12.30, 15.40)	13.40 (12.20, 15.60)	0.826	−0.074
PTT	29.10 (26.30, 33.70)	28.80 (26.10, 32.90)	32.35 (28.37, 40.52)	<0.001	0.257	31.20 (27.28, 39.23)	30.50 (26.70, 36.98)	32.00 (27.72, 41.03)	0.036	−0.124
Chloride, mEq/L	104.00 (101.00, 107.00)	104.00 (101.00, 107.00)	107.00 (103.00, 111.00)	<0.001	0.448	105.00 (101.00, 109.00)	105.00 (101.00, 109.00)	105.00 (101.00, 110.00)	0.810	0.022
Aniongap, mEq/L	14.00 (12.00, 16.00)	14.00 (12.00, 16.00)	12.00 (10.00, 15.00)	<0.001	−0.425	13.00 (11.00, 15.00)	13.00 (11.00, 15.00)	13.00 (11.00, 15.00)	0.919	−0.015
Bicarbonate, mEq/L	23.00 (21.00, 25.00)	23.00 (21.00, 25.00)	23.00 (21.00, 25.00)	0.478	0.054	23.00 (21.00, 25.00)	23.00 (21.00, 25.00)	23.00 (21.00, 25.00)	0.973	0.050
Calcium, mg/dL	8.70 (8.30, 9.10)	8.80 (8.30, 9.10)	8.50 (8.00, 9.00)	<0.001	−0.306	8.60 (8.10, 9.00)	8.50 (8.00, 8.90)	8.60 (8.10, 9.00)	0.417	0.005
Score										
SOFA	1.00 (0.00, 2.00)	0.00 (0.00, 2.00)	2.00 (1.00, 3.00)	<0.001	0.575	1.00 (0.00, 3.00)	1.00 (0.00, 3.00)	1.00 (0.00, 2.00)	0.919	−0.021
SAPSII	32.00 (25.00, 40.00)	31.00 (25.00, 40.00)	36.50 (31.00, 44.25)	<0.001	0.481	35.00 (29.00, 43.00)	35.00 (29.00, 43.00)	35.00 (30.00, 42.75)	0.597	0.017
GCS	15.00 (14.00, 15.00)	15.00 (14.00, 15.00)	15.00 (15.00, 15.00)	<0.001	0.069	15.00 (14.00, 15.00)	15.00 (14.00, 15.00)	15.00 (15.00, 15.00)	0.142	−0.002
Charlson Comorbidity Index	6.00 (4.00, 8.00)	6.00 (4.00, 8.00)	6.00 (4.75, 8.00)	0.508	0.043	7.00 (5.00, 9.00)	7.00 (5.00, 9.00)	7.00 (5.00, 8.75)	0.229	−0.095
Complication										
Myocardial Infarct, *n* (%)				<0.001					0.411	
No	2,945 (85.76)	2,683 (87.17)	262 (73.60)		−0.308	346 (72.69)	169 (71.01)	177 (74.37)		0.077
Yes	489 (14.24)	395 (12.83)	94 (26.40)		0.308	130 (27.31)	69 (28.99)	61 (25.63)		−0.077
Renal Disease, *n* (%)				<0.001					1.000	
No	2,850 (82.99)	2,583 (83.92)	267 (75.00)		−0.206	348 (73.11)	174 (73.11)	174 (73.11)		0.000
Yes	584 (17.01)	495 (16.08)	89 (25.00)		0.206	128 (26.89)	64 (26.89)	64 (26.89)		0.000
Liver Disease, *n* (%)				0.384					0.399	
No	3,288 (95.75)	2,944 (95.65)	344 (96.63)		0.054	463 (97.27)	233 (97.90)	230 (96.64)		−0.070
Yes	146 (4.25)	134 (4.35)	12 (3.37)		−0.054	13 (2.73)	5 (2.10)	8 (3.36)		0.070
Congestive Heart Failure, *n* (%)				<0.001					0.505	
No	2,643 (76.97)	2,402 (78.04)	241 (67.70)		−0.221	303 (63.66)	148 (62.18)	155 (65.13)		0.062
Yes	791 (23.03)	676 (21.96)	115 (32.30)		0.221	173 (36.34)	90 (37.82)	83 (34.87)		−0.062
Peripheral Vascular Disease, *n* (%)				<0.001					0.213	
No	2,993 (87.16)	2,737 (88.92)	256 (71.91)		−0.379	350 (73.53)	169 (71.01)	181 (76.05)		0.118
Yes	441 (12.84)	341 (11.08)	100 (28.09)		0.379	126 (26.47)	69 (28.99)	57 (23.95)		−0.118
Dementia, *n* (%)				0.017					0.793	
No	3,269 (95.2)	2,921 (94.90)	348 (97.75)		0.193	461 (96.85)	231 (97.06)	230 (96.64)		−0.023
Yes	165 (4.8)	157 (5.10)	8 (2.25)		−0.193	15 (3.15)	7 (2.94)	8 (3.36)		0.023
Chronic Pulmonary Disease, *n* (%)				<0.001					0.531	
No	2,859 (83.26)	2,595 (84.31)	264 (74.16)		−0.232	352 (73.95)	173 (72.69)	179 (75.21)		0.058
Yes	575 (16.74)	483 (15.69)	92 (25.84)		0.232	124 (26.05)	65 (27.31)	59 (24.79)		−0.058
Rheumatic Disease, *n* (%)				0.005					1.000	
No	3,332 (97.03)	2,995 (97.30)	337 (94.66)		−0.117	446 (93.7)	223 (93.70)	223 (93.70)		0.000
Yes	102 (2.97)	83 (2.70)	19 (5.34)		0.117	30 (6.3)	15 (6.30)	15 (6.30)		0.000
Peptic Ulcer Disease, *n* (%)				0.580					1.000	
No	3,384 (98.54)	3,032 (98.51)	352 (98.88)		0.035	470 (98.74)	235 (98.74)	235 (98.74)		0.000
Yes	50 (1.46)	46 (1.49)	4 (1.12)		−0.035	6 (1.26)	3 (1.26)	3 (1.26)		0.000
Diabetes, *n* (%)				0.008					0.850	
No	2,345 (68.29)	2,124 (69.01)	221 (62.08)		−0.143	294 (61.76)	146 (61.34)	148 (62.18)		0.017
Yes	1,089 (31.71)	954 (30.99)	135 (37.92)		0.143	182 (38.24)	92 (38.66)	90 (37.82)		−0.017
Hyperlipidemia, *n* (%)				0.001					0.646	
No	1734 (50.5)	1,583 (51.43)	151 (42.42)		−0.182	219 (46.01)	107 (44.96)	112 (47.06)		0.042
Yes	1700 (49.5)	1,495 (48.57)	205 (57.58)		0.182	257 (53.99)	131 (55.04)	126 (52.94)		−0.042
Atrial Fibrillation, *n* (%)				0.044					0.778	
No	2,174 (63.31)	1966 (63.87)	208 (58.43)		−0.110	289 (60.71)	143 (60.08)	146 (61.34)		0.026
Yes	1,260 (36.69)	1,112 (36.13)	148 (41.57)		0.110	187 (39.29)	95 (39.92)	92 (38.66)		−0.026
Hypertension, *n* (%)				<0.001					0.926	
No	2,525 (73.53)	2,337 (75.93)	188 (52.81)		−0.463	267 (56.09)	134 (56.30)	133 (55.88)		−0.008
Yes	909 (26.47)	741 (24.07)	168 (47.19)		0.463	209 (43.91)	104 (43.70)	105 (44.12)		0.008
AKI, *n* (%)				<0.001					0.631	
No	922 (26.85)	866 (28.14)	56 (15.73)		−0.341	84 (17.65)	44 (18.49)	40 (16.81)		−0.045
Yes	2,512 (73.15)	2,212 (71.86)	300 (84.27)		0.341	392 (82.35)	194 (81.51)	198 (83.19)		0.045
Treatment										
Ventilator, *n* (%)				<0.001					1.000	
No	1,237 (36.02)	1,206 (39.18)	31 (8.71)		−1.081	62 (13.03)	31 (13.03)	31 (13.03)		0.000
Yes	2,197 (63.98)	1872 (60.82)	325 (91.29)		1.081	414 (86.97)	207 (86.97)	207 (86.97)		0.000
CRRT, *n* (%)				0.001					0.805	
No	3,387 (98.63)	3,043 (98.86)	344 (96.63)		−0.124	459 (96.43)	229 (96.22)	230 (96.64)		0.023
Yes	47 (1.37)	35 (1.14)	12 (3.37)		0.124	17 (3.57)	9 (3.78)	8 (3.36)		−0.023

HR, Hazard Ratio; CI, confidence interval. BUN, blood urea nitrogen; RDW, red cell distribution width; WBC, white blood cell count; MCHC, mean corpuscular hemoglobin concentration; RDW, red cell distribution width; RBC, red blood cells; MCV, mean corpuscular volume; MCH, mean corpuscular hemoglobin; INR, international normalized ratio; PT, prothrombin time; PTT, partial thromboplastin time; SBP, systolic blood pressure; DBP, diastolic blood pressure; MBP, mean blood pressure; Resp rate, respiratory rate; SpO₂, peripheral oxygen saturation; AKI, acute kidney injury; CRRT, continuous renal replacement therapy; SOFA, sequential organ failure assessment; GCS, Glasgow Coma scale; SAPSII, simplified acute physiology score II.

### 3.2 Nitroglycerin and primary outcomes

Nitroglycerin use was significantly associated with 28-day mortality in ischemic stroke patients, both before and after propensity score matching (PSM). In the univariate Cox regression analysis, nitroglycerin use was significantly associated with lower 28-day mortality in ischemic stroke patients both before and after propensity score matching. Before PSM, the hazard ratio (HR) was 0.52 (95% CI: 0.38–0.71, *p* < 0.001), and after PSM, the association remained significant with an HR of 0.50 (95% CI, 0.32–0.79, *p* = 0.003) (Table 2).

**Table 2:** Association between nitroglycerin and clinical outcomes in stroke.

Variables	Before PSM		After PSM	
	HR (95% CI)	*P*	HR (95% CI)	*P*
Nitroglycerin				
No	1.00 (Reference)		1.00 (Reference)	
Yes	0.52 (0.38 ~ 0.71)	<0.001	0.50 (0.32 ~ 0.79)	0.003

HR, Hazard Ratio; CI, confidence interval; PSM, Propensity score matching.

Univariable Cox proportional hazards regression was performed to identify potential risk factors for 28-day mortality (Supplementary Table S3). This association was further evaluated using multivariable Cox models in both unmatched and matched cohorts (Table 3). Multivariate Cox regression analysis showed that nitroglycerin use was significantly associated with reduced 28-day mortality in ischemic stroke patients across all models before and after propensity score matching. Before PSM, the hazard ratios were 0.52 (*p* < 0.001) in Model 1, 0.55 (*p* < 0.001) in Model 2, 0.40 (*p* < 0.001) in Model 3, and 0.46 (*p* < 0.001) in Model 4. After PSM, the hazard ratios were 0.50 (*p* = 0.003) in Model 1, 0.51 (*p* = 0.003) in Model 2, 0.50 (*p* = 0.003) in Model 3, and 0.44 (*p* < 0.001) in Model 4. Variables in Model 3 and Model 4 were selected from univariable analyses, retaining only those with *p* < 0.05 (Supplementary Table S3).

**Table 3:** Association of nitroglycerin and the risk of 28-day mortality.

Variables	Before PSM								After PSM							
	Model 1		Model 2		Model 3		Model 4		Model 1		Model 2		Model 3		Model 4	
	HR (95%CI)	*P*	HR (95%CI)	*P*	HR (95%CI)	*P*	HR (95%CI)	*P*	HR (95%CI)	*P*	HR (95%CI)	*P*	HR (95%CI)	*P*	HR (95%CI)	*P*
Nitroglycerin																
No	1.00 (Reference)		1.00 (Reference)		1.00 (Reference)				1.00 (Reference)		1.00 (Reference)		1.00 (Reference)		1.00 (Reference)	
Yes	0.52 (0.38 ~ 0.71)	<0.001	0.55 (0.40 ~ 0.76)	<0.001	0.40 (0.29 ~ 0.55)	<0.001	0.46 (0.33 ~ 0.64)	<0.001	0.50 (0.32 ~ 0.79)	0.003	0.51 (0.33 ~ 0.80)	0.003	0.50 (0.32 ~ 0.78)	0.003	0.44 (0.27 ~ 0.71)	<0.001

HR, Hazard Ratio; CI, confidence interval.

Model 1: Crude.

Model 2: Adjust: gender, race, age.

Model 3: Adjust: gender, race, age, myocardial infarct, renal disease, liver disease, congestive heart failure, dementia, chronic pulmonary disease, hyperlipidemia, atrial fibrillation, hypertension, AKI, ventilator, CRRT.

Model 4: Adjust: gender, race, age, myocardial infarct, renal disease, liver disease, congestive heart failure, dementia, chronic pulmonary disease, hyperlipidemia, atrial fibrillation, hypertension, AKI, ventilator, CRRT, heart rate, resp rate, glucose, BUN, creatinine, WBC, MCHC, RDW, RBC, MCV, hemoglobin, hematocrit, INR, PT, anion gap, bicarbonate, calcium, SOFA, SAPSII, GCS, Charlson comorbidity index.

**Figure 3:**
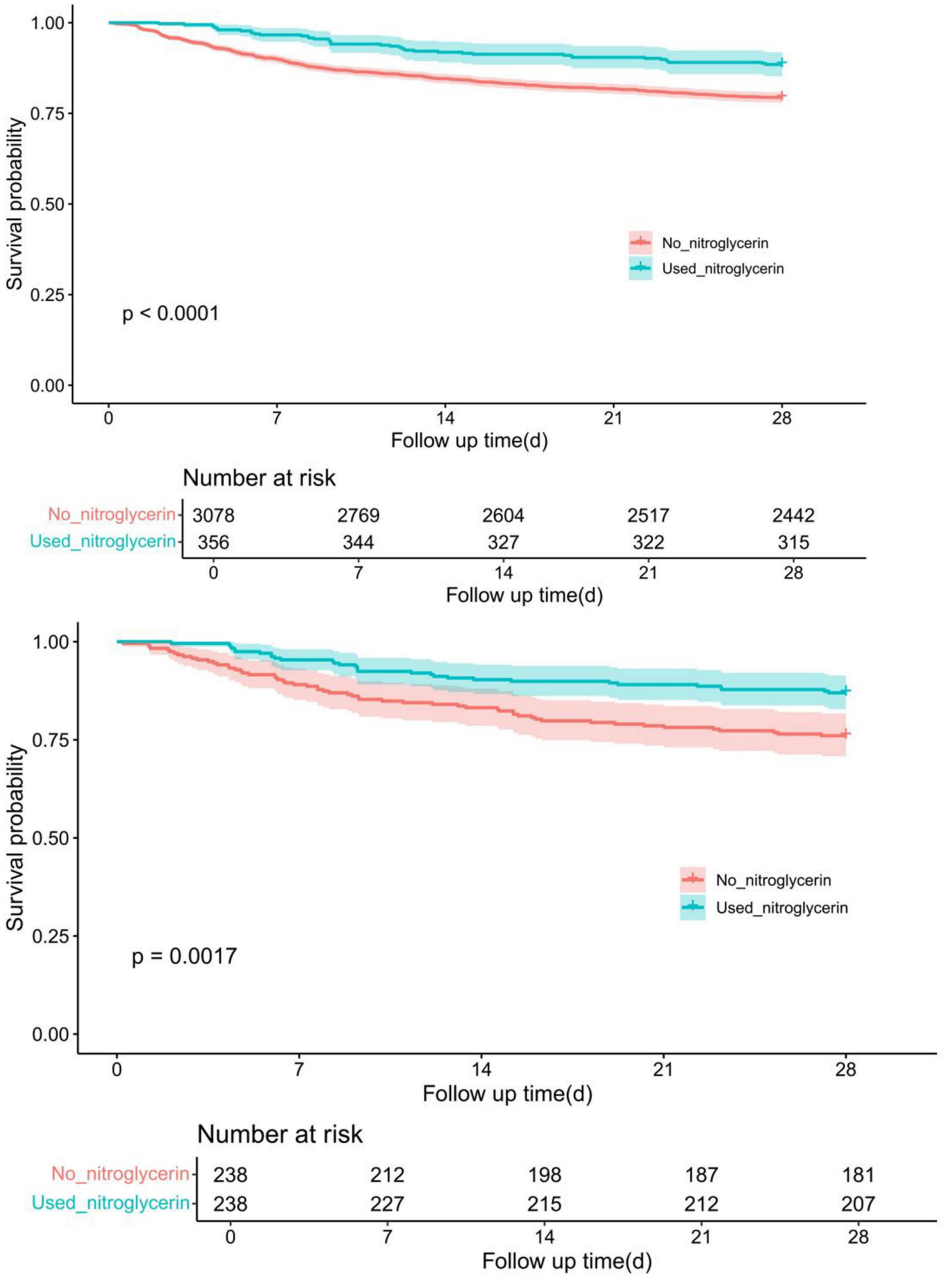
Comparison of Kaplan–Meier survival curves between nitroglycerin group and control group before and after PSM adjustment. The upper panel shows the survival difference between the two groups before PSM, while the lower panel presents the adjusted survival curves after 1:1 nearest-neighbor matching (caliper = 0.1).

Kaplan–Meier survival analysis revealed a significant difference in 28-day survival between patients who received nitroglycerin and those who did not. Before propensity score matching (PSM), the nitroglycerin group showed a significantly higher survival probability compared to the control group (*p* < 0.0001). After 1:1 nearest-neighbor matching with a caliper of 0.1, the survival advantage in the nitroglycerin group remained statistically significant (*p* = 0.0017) (Figure 3).

### 3.3 Dose of nitroglycerin and outcomes

A ROC curve analysis was conducted based on the cumulative intravenous nitroglycerin dose, and a cutoff value of 100.3 mg was determined as the threshold. Patients were stratified into two groups: those receiving ≤100.3 mg (*n* = 145) and >100.3 mg (*n* = 175). The same propensity score matching (PSM) strategy used to compare nitroglycerin users and non-users was applied to evaluate outcomes across dosage groups. Detailed results are provided in Supplementary Table S4.

**Figure 4:**
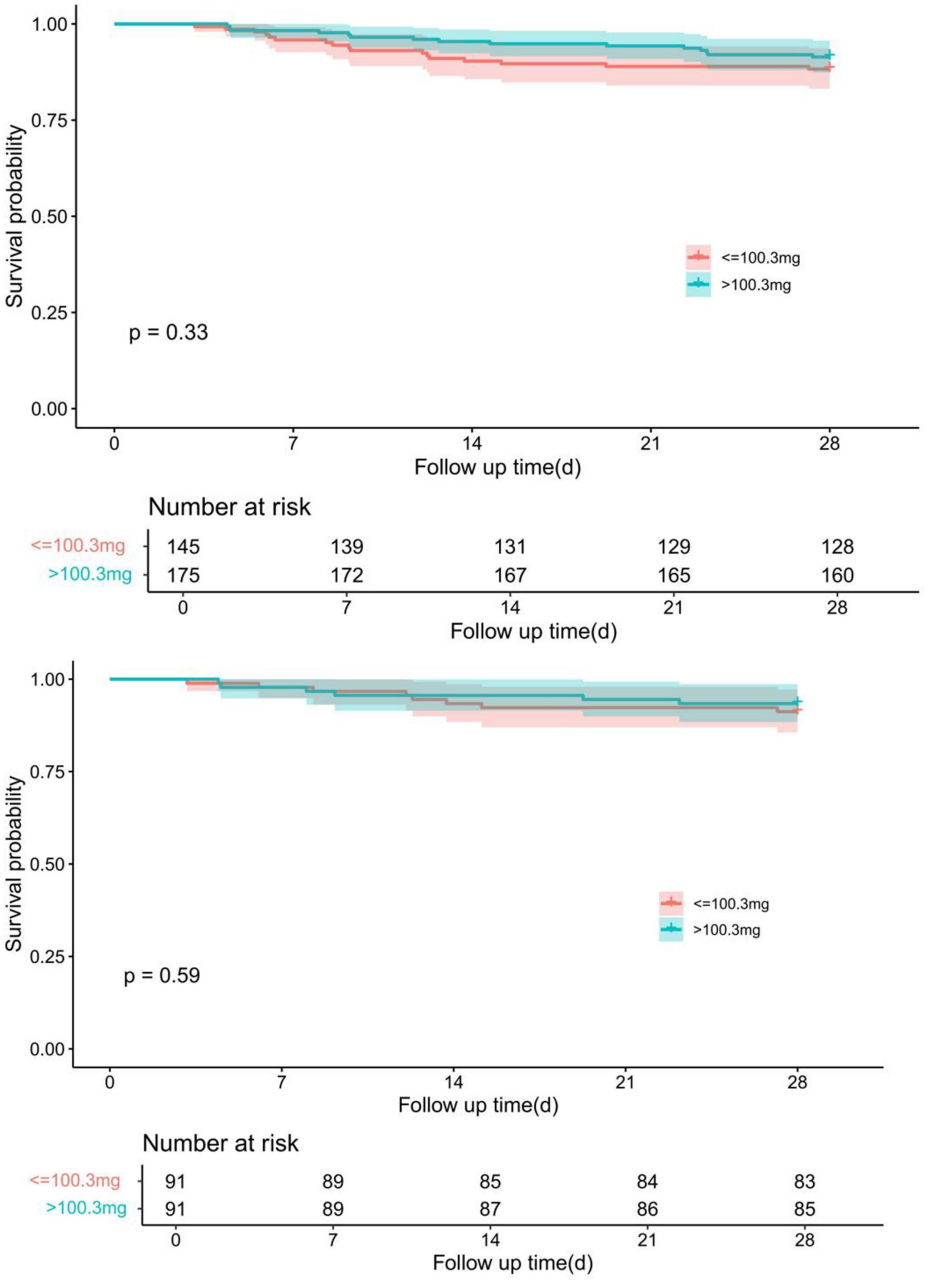
Comparison of Kaplan–Meier survival curves for different dose groups of nitroglycerin before and after PSM adjustment. The upper panel shows the survival difference between groups prior to PSM, while the lower panel demonstrates the adjusted survival curves after 1:1 nearest-neighbor matching with a caliper of 0.1.

Kaplan–Meier survival analysis showed no significant difference in 28-day survival between patients receiving low-dose (≤100.3 mg) and high-dose (>100.3 mg) nitroglycerin. Before propensity score matching (PSM), the survival probability was similar between the two groups (*p* = 0.33). After 1:1 nearest-neighbor matching with a caliper of 0.1, the difference remained non-significant (*p* = 0.59) (Figure 4).

### 3.4 Association of nitroglycerin administration characteristics with 28-day in-hospital mortality

In this propensity-score-matched cohort of patients classified according to whether they received nitroglycerin within the first 28 days of hospitalization, we evaluated the association between nitroglycerin administration route, initiation timing and duration of use with 28-day in-hospital mortality. The results showed that only IV DRIP administration was significantly associated with reduced 28-day mortality (Model 4: HR = 0.32, 95% CI 0.19–0.57, *p* < 0.001), whereas sublingual or transdermal/oral routes were not statistically significant (Table 4). Regarding timing, initiating nitroglycerin within 24 h of admission was the only time window linked to decreased mortality (Model 4: HR = 0.29, 95% CI 0.16–0.54, *p* < 0.001), while initiation after day 1 showed no significant effect (Table 5). Similarly, a treatment duration of 1–3 days was significantly associated with lower 28-day mortality (Model 4: HR = 0.30, 95% CI 0.16–0.60, *p* < 0.001) (Table 6). In summary, IV administration, early initiation within 24 h and a 1–3-day course were each independently associated with reduced 28-day in-hospital mortality.

**Table 4:** The effect of different nitroglycerin administration routes on mortality risk.

Nitroglycerin route	Model 1		Model 2		Model 3		Model 4	
Variables	*P*	HR (95%CI)	*P*	HR (95%CI)	*P*	HR (95%CI)	*P*	HR (95%CI)
No nitroglycerin		1.00 (Reference)		1.00 (Reference)		1.00 (Reference)		1.00 (Reference)
IV DRIP	<0.001	0.36 (0.21 ~ 0.60)	<0.001	0.37 (0.22 ~ 0.63)	<0.001	0.37 (0.22 ~ 0.62)	<0.001	0.32 (0.19 ~ 0.57)
SL	0.488	1.30 (0.62 ~ 2.72)	0.812	1.10 (0.52 ~ 2.33)	0.943	0.97 (0.45 ~ 2.10)	0.736	1.16 (0.50 ~ 2.67)
TD/PO/TP	0.318	1.68 (0.61 ~ 4.62)	0.461	1.47 (0.53 ~ 4.10)	0.4	1.58 (0.54 ~ 4.61)	0.572	0.70 (0.21 ~ 2.38)

HR, Hazard Ratio; CI, confidence interval.

Model 1: Crude.

Model 2: Adjust: gender, race, age.

Model 3: Adjust: gender, race, age, myocardial infarct, renal disease, liver disease, congestive heart failure, dementia, chronic pulmonary disease, hyperlipidemia, atrial fibrillation, hypertension, AKI, ventilator, CRRT.

Model 4: Adjust: gender, race, age, myocardial infarct, renal disease, liver disease, congestive heart failure, dementia, chronic pulmonary disease, hyperlipidemia, atrial fibrillation, hypertension, AKI, ventilator, CRRT, heart rate, resp rate, glucose, BUN, creatinine, WBC, MCHC, RDW, RBC, MCV, hemoglobin, hematocrit, INR, PT, anion gap, bicarbonate, calcium, SOFA, SAPSII, GCS, Charlson comorbidity index.

**Table 5:** The association between nitroglycerin administration timing and mortality risk.

Administration time group	Model 1		Model 2		Model 3		Model 4	
Variables	*P*	HR (95%CI)	*P*	HR (95%CI)	*P*	HR (95%CI)	*P*	HR (95%CI)
No nitroglycerin		1.00 (Reference)		1.00 (Reference)		1.00 (Reference)		1.00 (Reference)
<1d	<0.001	0.31 (0.17 ~ 0.55)	<0.001	0.31 (0.17 ~ 0.56)	<0.001	0.32 (0.17 ~ 0.57)	<0.001	0.29 (0.16 ~ 0.54)
1-3d	0.936	1.03 (0.56 ~ 1.87)	0.982	0.99 (0.54 ~ 1.82)	0.738	0.90 (0.48 ~ 1.68)	0.416	0.75 (0.37 ~ 1.51)
≥4d	0.775	1.16 (0.42 ~ 3.19)	0.554	1.36 (0.49 ~ 3.78)	0.777	1.17 (0.40 ~ 3.37)	0.921	0.94 (0.27 ~ 3.22)

HR, Hazard Ratio; CI, confidence interval.

Model 1: Crude.

Model 2: Adjust: gender, race, age.

Model 3: Adjust: gender, race, age, myocardial infarct, renal disease, liver disease, congestive heart failure, dementia, chronic pulmonary disease, hyperlipidemia, atrial fibrillation, hypertension, AKI, ventilator, CRRT.

Model 4: Adjust: gender, race, age, myocardial infarct, renal disease, liver disease, congestive heart failure, dementia, chronic pulmonary disease, hyperlipidemia, atrial fibrillation, hypertension, AKI, ventilator, CRRT, heart rate, resp rate, glucose, BUN, creatinine, WBC, MCHC, RDW, RBC, MCV, hemoglobin, hematocrit, INR, PT, anion gap, bicarbonate, calcium, SOFA, SAPSII, GCS, Charlson comorbidity index.

**Table 6:** The association between duration of nitroglycerin use and mortality risk.

Duration of use group	Model 1		Model 2		Model 3		Model 4	
Variables	*P*	HR (95%CI)	*P*	HR (95%CI)	*P*	HR (95%CI)	*P*	HR (95%CI)
No nitroglycerin		1.00 (Reference)		1.00 (Reference)		1.00 (Reference)		1.00 (Reference)
<1d	0.250	1.54 (0.74 ~ 3.24)	0.631	1.20 (0.56 ~ 2.57)	0.562	1.26 (0.58 ~ 2.75)	0.670	0.83 (0.35 ~ 1.98)
1–3d	<0.001	0.30 (0.16 ~ 0.56)	<0.001	0.31 (0.17 ~ 0.57)	<0.001	0.29 (0.16 ~ 0.55)	<0.001	0.30 (0.16 ~ 0.60)
≥4d	0.206	0.66 (0.35 ~ 1.26)	0.343	0.73 (0.38 ~ 1.40)	0.396	0.75 (0.38 ~ 1.46)	0.068	0.52 (0.26 ~ 1.05)

HR, Hazard Ratio; CI, confidence interval.

Model 1: Crude.

Model 2: Adjust: gender, race, age.

Model 3: Adjust: gender, race, age, myocardial infarct, renal disease, liver disease, congestive heart failure, dementia, chronic pulmonary disease, hyperlipidemia, atrial fibrillation, hypertension, AKI, ventilator, CRRT.

Model 4: Adjust: gender, race, age, myocardial infarct, renal disease, liver disease, congestive heart failure, dementia, chronic pulmonary disease, hyperlipidemia, atrial fibrillation, hypertension, AKI, ventilator, CRRT, heart rate, resp rate, glucose, BUN, creatinine, WBC, MCHC, RDW, RBC, MCV, hemoglobin, hematocrit, INR, PT, anion gap, bicarbonate, calcium, SOFA, SAPSII, GCS, Charlson comorbidity index.

### 3.5 Subgroup analysis

**Figure 5:**
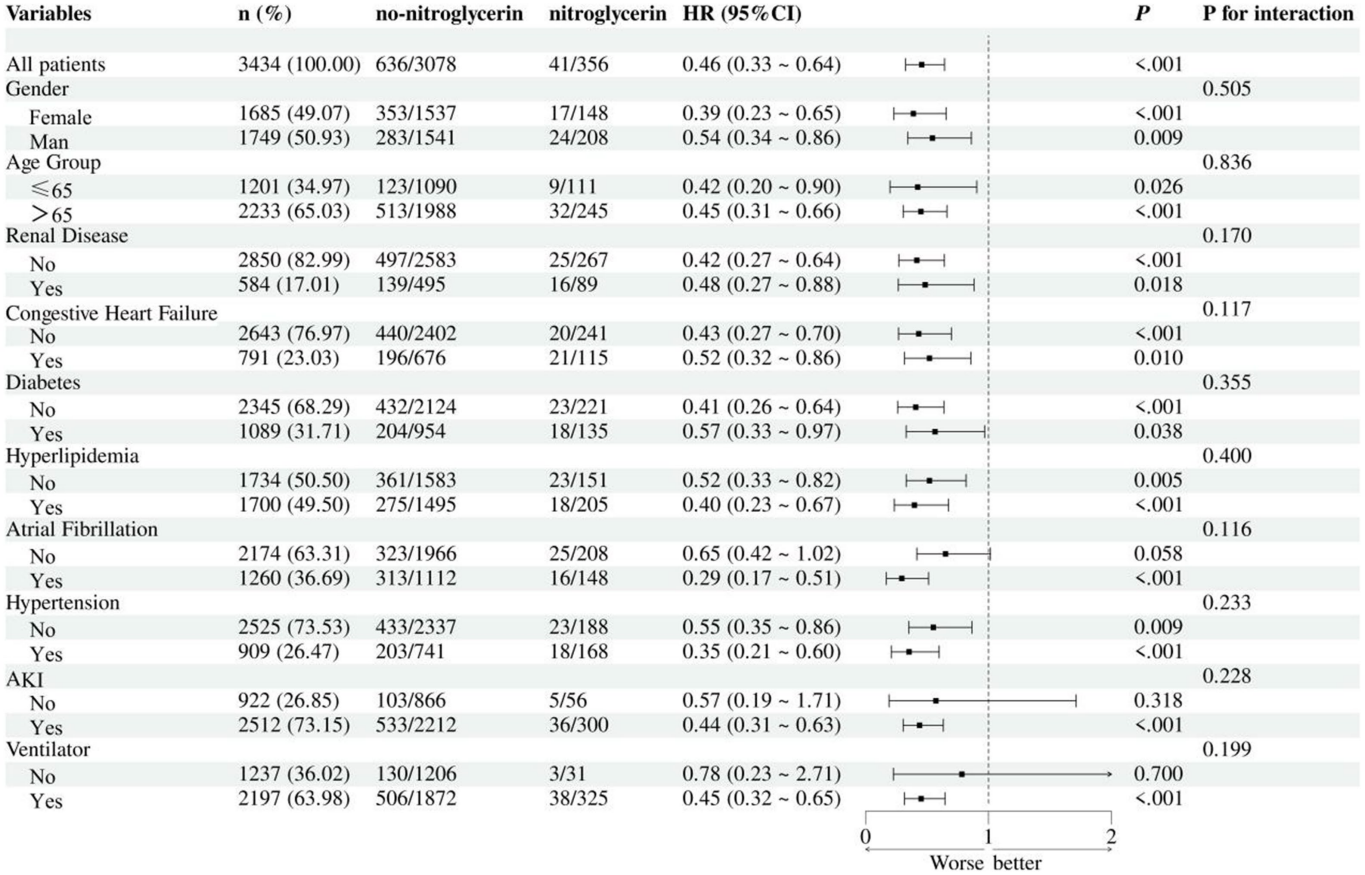
Subgroup analysis of nitroglycerin impact on 28-day ICU mortality.

In the subgroup analysis, nitroglycerin use was associated with reduced 28-day mortality across multiple clinical subgroups (Figure 5). The survival benefit remained significant in both female and male patients, as well as in those with or without renal disease, heart failure, diabetes, or hyperlipidemia (all *p* < 0.05). Although the effect appeared more pronounced in certain groups, such as female patients and those with diabetes, no statistically significant interactions were observed.

## 4 Discussion

This retrospective cohort study of 3,434 ischemic stroke patients found that nitroglycerin use was significantly associated with reduced 28-day in-hospital mortality. The drug was administered via IV drip, sublingual, or transcutaneous routes. After 1:1 propensity score matching, covariate balance was achieved. Cox regression and Kaplan–Meier analyses showed significantly improved survival in the nitroglycerin group before (*p* < 0.0001) and after matching (*p* = 0.0017). Further analysis revealed that only IV drip administration significantly reduced mortality risk (HR = 0.32, *p* < 0.001), while sublingual and transdermal/oral routes showed no significant effect. Initiating treatment within 24 h (HR = 0.29) and a duration of 1–3 days (HR = 0.30) were also associated with lower mortality. These findings suggest that early, short-term intravenous nitroglycerin may offer a survival benefit in ischemic stroke patients.

Nitroglycerin exerts its effects through NO-mediated sGC activation, inducing vasodilation while also reducing oxidative stress and cellular damage (18, 19). Recent studies have highlighted the protective effects of NTG in ischemia–reperfusion injury and myocardial protection through CGRP signaling, suggesting broader therapeutic potential beyond angina management. Additionally, NTG may help mitigate nitrate tolerance and enhance overall clinical efficacy (20). Nitroglycerin is primarily known for its effectiveness in treating angina pectoris due to its vasodilatory properties, which improve blood flow by releasing NO (21). Moreover, NTG has shown potential in oncology, as its nitric oxide (NO)-releasing properties may help overcome tumor resistance and enhance the efficacy of cancer therapies (22). Additionally, NTG has been investigated in the context of cerebral ischemia; however, outcomes in ischemic stroke therapy remain inconsistent, highlighting the need for further research to clarify its neuroprotective potential (23).

Although studies have examined the use of nitroglycerin in ischemic stroke, there is still considerable uncertainty about its efficacy. RIGHT-2 Trial Suggests Prehospital nitroglycerin Therapy Fails to Improve Prognosis in Ischemic Stroke Patients and May Be Harmful to Certain Subgroups (24). Thus, current evidence supports the potential of nitroglycerin in specific populations, but its broad applicability needs to be validated by further studies.

This study found that the therapeutic efficacy of nitroglycerin in acute ischemic stroke varies due to multiple factors. Timing of administration is a key determinant: studies have shown that using NTG within 2–6 h after stroke onset may improve functional outcomes, whereas administration during the ultra-acute phase (within 1 h) may be ineffective or even harmful (10). The baseline condition of patients also plays an important role. For example, patients in the RIGHT-2 trial were generally older, functionally dependent prior to stroke, and exhibited radiological signs of cerebral frailty, making them potentially more sensitive to blood pressure reduction and thereby affecting cerebral perfusion. Meanwhile, the degree of carotid artery stenosis also influences the efficacy of NTG; analysis from the ENOS trial suggested that NTG may lead to better outcomes in patients with ≥70% stenosis (11). Duration of treatment and patient adherence are also important considerations. In-hospital studies typically involve a complete treatment course, whereas prehospital use is often subject to interruption, which may reduce efficacy. In summary, the therapeutic effect of NTG depends on the time window after stroke onset, individual perfusion status, and underlying health conditions. Further research is needed to identify appropriate patient subgroups and optimize treatment strategies.

Nitroglycerin is widely used in the ICU to manage critical conditions such as septic shock, acute respiratory distress syndrome (ARDS), and acute hypertensive heart failure (25, 26). In patients with septic shock, NTG improves hemodynamics, reduces inflammation, and shortens ICU and hospital stays by enhancing microcirculation and lowering pulmonary vascular resistance. NTG also helps reduce blood pressure and prevent ICU admission in patients with hypertensive heart failure. Additionally, NTG has shown effectiveness in resuscitating patients with refractory pulseless electrical activity (PEA) due to coronary spasms, and is also used in neonatal ICUs to treat ischemic injuries in preterm infants (27, 28). These diverse applications demonstrate NTG’s versatility in critical care.

In the pathophysiology of ischemic stroke, the nitric oxide (NO) pathway exerts multiple regulatory effects that are dependent on the source, timing, and treatment modality. Following ischemic stroke, intracellular calcium overload activates nitric oxide synthase (NOS), which catalyzes the production of NO from L-arginine. Excessive NO generated by neuronal NOS (nNOS) exacerbates oxidative stress and neuronal injury by producing reactive species such as peroxynitrite, whereas endothelial NOS (eNOS) promotes vasodilation and improves cerebral perfusion during the early phase of ischemic stroke, thereby exerting neuroprotective effects. In addition, inducible NOS (iNOS) is upregulated in response to inflammation, producing large amounts of NO that contribute to inflammatory injury during the later stages of ischemic stroke (29). Some studies have further emphasized that nNOS and iNOS are key mediators of ischemic stroke injury, whereas eNOS is closely related to the protection of vascular function (30). Recent studies have demonstrated that treatment with inhaled nitric oxide (iNO) restores impaired cGMP signaling following ischemia, inhibits the expression of adhesion molecules such as ICAM-1 and VCAM-1, and reduces leukocyte-endothelial interactions and the upregulation of pro-inflammatory mediators in brain tissue, thereby significantly attenuating inflammation and secondary injury. Thus, the NO signaling pathway is not only a key mechanism in ischemic stroke pathophysiology but also represents a promising therapeutic target for time-dependent, precision interventions (31).

Moreover, preclinical and pilot clinical studies have shown that exogenous NO delivery—particularly via inhaled nitric oxide (iNO)—may improve endothelial function and microvascular perfusion in ischemic stroke, although evidence remains limited due to small sample sizes and lack of standardized outcomes (32). In parallel, novel NO-generating technologies using redox-promoted CO₂ adsorption offer a tablet-free, air-derived source of therapeutic NO, enabling controllable, low-cost delivery that may hold promise in low-resource or emergency settings (33). These emerging strategies further support the potential of NO-based therapy as a feasible and innovative avenue for ischemic stroke intervention.

Nitroglycerin exerts neuroprotective effects in cerebral ischemia through the nitric oxide (NO)–cGMP–PKG signaling pathway by reducing oxidative stress, apoptosis, and endoplasmic reticulum (ER) stress. In a rat model, NTG was shown to enhance glucose metabolism, reduce reactive oxygen species (ROS) and lactate levels, and improve neurological outcomes—effects that were dependent on PKG activation (34). Additionally, NTG-induced preconditioning mimics ischemic preconditioning, modulating mitochondrial responses and reducing free radical damage, suggesting a potential therapeutic role in ischemic stroke (35).

Although NTG lowers blood pressure, which theoretically may reduce cerebral perfusion and exacerbate infarct expansion, several proposed mechanisms suggest potential neuroprotective benefits. Thus, the net impact of NTG may reflect a balance between its systemic hypotensive action and its cerebral protective effects. Further studies are warranted to clarify these competing influences in different stroke subtypes and blood pressure profiles.

Subgroup analyses further confirmed the survival benefit of nitroglycerin across diverse patient populations. The association with reduced 28-day mortality remained statistically significant in both female and male patients, as well as in subgroups stratified by age, renal function, heart failure status, diabetes, hyperlipidemia, and ventilator use. Although the protective effect appeared more pronounced in certain populations, such as females and those with diabetes or hyperlipidemia, no statistically significant interactions were detected, indicating that the observed benefit was generally consistent across subgroups. These findings suggest that the effect of nitroglycerin may be broadly applicable in ischemic stroke patients regardless of baseline characteristics or comorbidities, and not limited to specific high-risk populations. The absence of interaction effects also supports the robustness of the overall association and reduces concern about effect modification.

This study has several important limitations. First, the MIMIC-IV database lacks key ischemic stroke-specific clinical variables, including measures of ischemic stroke severity (e.g., NIH Stroke Scale), etiological classification (e.g., TOAST), infarct location, and pre-stroke functional status. The absence of these variables may compromise baseline risk stratification and limit adjustment for important confounders. Second, the database does not capture standardized functional or neurological outcome measures, such as the modified Rankin Scale (mRS), Barthel Index, or NIHSS at discharge and follow-up, nor does it include recurrent stroke data or long-term functional recovery. As a result, our study was limited to 28-day mortality—a relatively coarse endpoint that may fail to detect more subtle but clinically meaningful benefits of nitroglycerin, such as neuroprotection or improved functional outcomes. Third, since MIMIC-IV only includes ICU admissions, patients managed in specialized ischemic stroke units are not represented, and ICU admission criteria are not standardized. This may introduce selection bias and limit the generalizability of our findings to the broader population of ischemic stroke patients. Lastly, the relatively small number of patients with isolated ischemic stroke as the primary diagnosis raises the possibility of model overfitting in multivariable analyses, a known limitation in prior MIMIC-based ischemic stroke studies. These constraints underscore the need for prospective, multi-center research with detailed ischemic stroke phenotyping, functional outcome tracking, and standardized inclusion criteria to validate our findings.

## 5 Conclusion

This retrospective study found that nitroglycerin use was significantly associated with reduced 28-day in-hospital mortality in patients with ischemic stroke. This association remained robust after propensity score matching and was confirmed through multivariate Cox regression, Kaplan–Meier survival analysis, and subgroup analyses, with no significant interaction effects across clinical subgroups. Further stratified analyses indicated that the survival benefit was specific to IV drip administration, particularly when initiated within 24 h of admission and maintained for 1–3 days. No mortality difference was observed between low- and high-dose groups. These findings suggest a potential protective effect of early, short-term intravenous nitroglycerin in acute ischemic stroke, warranting further prospective validation.

## Data Availability

The datasets presented in this study can be found in online repositories. The names of the repository/repositories and accession number(s) can be found: https://physionet.org/content/mimiciv/2.0/.
