# Supplementary material for: Impact of nitroglycerin on 28-day mortality in ischemic stroke patients: a retrospective cohort study using the MIMIC-IV database
Source: Front Neurol. 2025 Sep 12;16:1577700. doi: 10.3389/fneur.2025.1577700 (PMC12464029; doi:10.3389/fneur.2025.1577700)
Supplement: Supplementary file 1 [file Data_Sheet_1.docx]

Table S1.

A)Nitroglycerin route of use statistics

| Route | Numbers | Percentage |
| --- | --- | --- |
| IV DRIP | 320 | 89.8% |
| SL | 26 | 7.3% |
| TP | 8 | 2.2% |
| PO | 1 | 0.1% |
| TD | 1 | 0.1% |

IV DRIP,Intravenous Drip;SL,Sublingual;TP,Transpulmonary;PO,Per Oral;TD,Transdermal

B)Time from ICU Admission to Nitroglycerin Administration

| Administration time | Numbers | Percentage |
| --- | --- | --- |
| ＜1 | 282 | 79.2% |
| 1 | 35 | 9.8% |
| 2 | 17 | 4.8% |
| 3 | 7 | 2.0% |
| 4 | 3 | 0.8% |
| 5 | 2 | 0.6% |
| 6 | 1 | 0.3% |
| 7 | 1 | 0.3% |
| 8 | 3 | 0.8% |
| 9 | 2 | 0.6% |
| 12 | 2 | 0.6% |
| 13 | 1 | 0.3% |

C)The duration of nitroglycerin use within 28 days of ICU admission.

| Usage time | Numbers | Percentage |
| --- | --- | --- |
| ＜1 | 25 | 7.0% |
| 1 | 97 | 27.2% |
| 2 | 68 | 19.1% |
| 3 | 58 | 16.3% |
| 4 | 32 | 9.0% |
| 5 | 19 | 5.3% |
| 6 | 16 | 4.5% |
| 7 | 9 | 2.5% |
| 8 | 5 | 1.4% |
| 9 | 8 | 2.2% |
| 10 | 6 | 1.7% |
| 11 | 2 | 0.6% |
| 12 | 4 | 1.1% |
| 15 | 1 | 0.3% |
| 17 | 1 | 0.3% |
| 18 | 1 | 0.3% |
| 20 | 1 | 0.3% |
| 21 | 1 | 0.3% |
| 26 | 1 | 0.3% |
| 27 | 1 | 0.3% |

Table S2.Baseline characteristics of patients at 28-day survival and 28-day mortality.

| Variables | Total (n = 3434) | 28-day survival (n = 2757) | 28-day mortality (n = 677) | *P* |
| --- | --- | --- | --- | --- |
|  |  |  |  |  |
| Age | 71.00 (61.00, 82.00) | 70.00 (59.00, 80.00) | 79.00 (68.00, 87.00) | <0.001 |
| Gender, n(%) |  |  |  | 0.001 |
| Female | 1685 (49.07) | 1315 (47.70) | 370 (54.65) |  |
| Man | 1749 (50.93) | 1442 (52.30) | 307 (45.35) |  |
| Race, n(%) |  |  |  | <0.001 |
| White | 2153 (62.70) | 1782 (64.64) | 371 (54.80) |  |
| Black | 342 (9.96) | 284 (10.30) | 58 (8.57) |  |
| Others | 939 (27.34) | 691 (25.06) | 248 (36.63) |  |
| **Vital signs** |  |  |  |  |
| Heart Rate,bpm | 80.00 (69.00, 93.00) | 79.00 (69.00, 91.00) | 85.00 (72.00, 100.00) | <0.001 |
| SBP,mmHg | 139.00 (122.00, 155.00) | 139.00 (123.00, 155.00) | 139.00 (119.00, 156.00) | 0.447 |
| DBP,mmHg | 75.00 (64.00, 89.00) | 75.00 (64.00, 89.00) | 75.00 (63.00, 87.00) | 0.188 |
| MBP,mmHg | 93.00 (81.00, 106.00) | 93.00 (82.00, 106.00) | 93.00 (80.00, 104.00) | 0.096 |
| Resp Rate,bpm | 18.00 (15.00, 21.00) | 18.00 (15.00, 21.00) | 19.00 (16.00, 23.00) | <0.001 |
| Temperature,℃ | 36.72 (36.50, 37.00) | 36.72 (36.50, 37.00) | 36.67 (36.44, 37.06) | 0.079 |
| SpO2,% | 98.00 (96.00, 100.00) | 98.00 (96.00, 100.00) | 98.00 (96.00, 100.00) | 0.013 |
| **Laboratory index** |  |  |  |  |
| Glucose,mg/dL | 125.00 (103.00, 160.00) | 122.00 (101.00, 155.00) | 139.00 (115.00, 184.00) | <0.001 |
| BUN,mg/dL | 17.00 (12.00, 24.00) | 16.00 (12.00, 22.00) | 21.00 (15.00, 32.00) | <0.001 |
| Potassium,mEq/L | 4.00 (3.70, 4.40) | 4.00 (3.70, 4.40) | 4.10 (3.70, 4.60) | <0.001 |
| Sodium,mEq/L | 139.00 (137.00, 142.00) | 139.00 (137.00, 141.00) | 139.00 (136.00, 142.00) | 0.268 |
| Creatinine,mEq/L | 0.90 (0.70, 1.20) | 0.90 (0.70, 1.10) | 1.00 (0.80, 1.40) | <0.001 |
| WBC,K/uL | 9.70 (7.50, 12.90) | 9.50 (7.40, 12.40) | 11.50 (8.60, 15.30) | <0.001 |
| MCHC,% | 33.00 (32.00, 33.90) | 33.00 (32.10, 34.00) | 32.70 (31.60, 33.60) | <0.001 |
| RDW,% | 13.80 (13.10, 14.80) | 13.70 (13.00, 14.70) | 14.30 (13.50, 15.90) | <0.001 |
| RBC,M/uL | 4.02 (3.49, 4.49) | 4.05 (3.56, 4.51) | 3.86 (3.28, 4.39) | <0.001 |
| Platelet,K/uL | 209.00 (164.00, 266.00) | 209.00 (165.00, 264.00) | 209.00 (158.00, 277.00) | 0.832 |
| MCV,fL | 91.00 (87.00, 95.00) | 91.00 (87.00, 94.00) | 92.00 (87.00, 96.00) | 0.001 |
| MCH,pg | 30.10 (28.60, 31.40) | 30.10 (28.70, 31.40) | 30.10 (28.50, 31.40) | 0.901 |
| Hematocrit,% | 12.00 (10.30, 13.50) | 12.10 (10.40, 13.60) | 11.50 (9.90, 13.00) | <0.001 |
| Hemoglobin,g/dL | 36.40 (31.70, 40.50) | 36.80 (32.00, 40.60) | 35.40 (30.60, 39.70) | <0.001 |
| INR | 1.20 (1.10, 1.30) | 1.20 (1.10, 1.30) | 1.20 (1.10, 1.40) | <0.001 |
| PT | 12.80 (11.80, 14.38) | 12.60 (11.70, 14.10) | 13.40 (12.10, 15.40) | <0.001 |
| PTT | 29.10 (26.30, 33.70) | 29.10 (26.40, 33.50) | 29.30 (25.80, 33.90) | 0.412 |
| Chloride,mEq/L | 104.00 (101.00, 107.00) | 104.00 (101.00, 107.00) | 104.00 (101.00, 107.00) | 0.415 |
| Aniongap,mEq/L | 14.00 (12.00, 16.00) | 14.00 (12.00, 16.00) | 15.00 (13.00, 17.00) | <0.001 |
| Bicarbonate,mEq/L | 23.00 (21.00, 25.00) | 23.00 (21.00, 25.00) | 22.00 (20.00, 25.00) | <0.001 |
| Calcium,mg/dL | 8.70 (8.30, 9.10) | 8.80 (8.30, 9.20) | 8.50 (8.10, 9.00) | <0.001 |
| **Score** |  |  |  |  |
| SOFA | 1.00 (0.00, 2.00) | 0.00 (0.00, 2.00) | 1.00 (0.00, 2.00) | <0.001 |
| SAPSII | 32.00 (25.00, 40.00) | 30.00 (24.00, 37.00) | 41.00 (35.00, 50.00) | <0.001 |
| GCS | 15.00 (14.00, 15.00) | 15.00 (14.00, 15.00) | 15.00 (12.00, 15.00) | <0.001 |
| Charlson Comorbidity Index | 6.00 (4.00, 8.00) | 6.00 (4.00, 8.00) | 8.00 (6.00, 9.00) | <0.001 |
| **Complication** |  |  |  |  |
| Myocardial Infarct, n(%) |  |  |  | <0.001 |
| No | 2945 (85.76) | 2397 (86.94) | 548 (80.95) |  |
| Yes | 489 (14.24) | 360 (13.06) | 129 (19.05) |  |
| Renal Disease, n(%) |  |  |  | <0.001 |
| No | 2850 (82.99) | 2328 (84.44) | 522 (77.10) |  |
| Yes | 584 (17.01) | 429 (15.56) | 155 (22.90) |  |
| Liver Disease, n(%) |  |  |  | 0.009 |
| No | 3288 (95.75) | 2652 (96.19) | 636 (93.94) |  |
| Yes | 146 (4.25) | 105 (3.81) | 41 (6.06) |  |
| Congestive Heart Failure, n(%) |  |  |  | <0.001 |
| No | 2643 (76.97) | 2183 (79.18) | 460 (67.95) |  |
| Yes | 791 (23.03) | 574 (20.82) | 217 (32.05) |  |
| Peripheral Vascular Disease, n(%) |  |  |  | 0.904 |
| No | 2993 (87.16) | 2402 (87.12) | 591 (87.30) |  |
| Yes | 441 (12.84) | 355 (12.88) | 86 (12.70) |  |
| Dementia, n(%) |  |  |  | <0.001 |
| No | 3269 (95.20) | 2651 (96.16) | 618 (91.29) |  |
| Yes | 165 (4.80) | 106 (3.84) | 59 (8.71) |  |
| Chronic Pulmonary Disease, n(%) |  |  |  | 0.018 |
| No | 2859 (83.26) | 2316 (84.00) | 543 (80.21) |  |
| Yes | 575 (16.74) | 441 (16.00) | 134 (19.79) |  |
| Rheumatic Disease, n(%) |  |  |  | 0.822 |
| No | 3332 (97.03) | 2676 (97.06) | 656 (96.90) |  |
| Yes | 102 (2.97) | 81 (2.94) | 21 (3.10) |  |
| Peptic Ulcer Disease, n(%) |  |  |  | 0.682 |
| No | 3384 (98.54) | 2718 (98.59) | 666 (98.38) |  |
| Yes | 50 (1.46) | 39 (1.41) | 11 (1.62) |  |
| Diabetes, n(%) |  |  |  | 0.501 |
| No | 2345 (68.29) | 1890 (68.55) | 455 (67.21) |  |
| Yes | 1089 (31.71) | 867 (31.45) | 222 (32.79) |  |
| Hyperlipidemia, n(%) |  |  |  | <0.001 |
| No | 1734 (50.50) | 1350 (48.97) | 384 (56.72) |  |
| Yes | 1700 (49.50) | 1407 (51.03) | 293 (43.28) |  |
| Atrial Fibrillation, n(%) |  |  |  | <0.001 |
| No | 2174 (63.31) | 1826 (66.23) | 348 (51.40) |  |
| Yes | 1260 (36.69) | 931 (33.77) | 329 (48.60) |  |
| Hypertension, n(%) |  |  |  | <0.001 |
| No | 2525 (73.53) | 2069 (75.05) | 456 (67.36) |  |
| Yes | 909 (26.47) | 688 (24.95) | 221 (32.64) |  |
| AKI, n(%) |  |  |  | <0.001 |
| No | 922 (26.85) | 814 (29.52) | 108 (15.95) |  |
| Yes | 2512 (73.15) | 1943 (70.48) | 569 (84.05) |  |
| Treatment |  |  |  |  |
| Nitroglycerin, n(%) |  |  |  | <0.001 |
| No | 3078 (89.63) | 2442 (88.57) | 636 (93.94) |  |
| Yes | 356 (10.37) | 315 (11.43) | 41 (6.06) |  |
| Ventilator, n(%) |  |  |  | <0.001 |
| No | 1237 (36.02) | 1104 (40.04) | 133 (19.65) |  |
| Yes | 2197 (63.98) | 1653 (59.96) | 544 (80.35) |  |
| CRRT, n(%) |  |  |  | 0.004 |
| No | 3387 (98.63) | 2727 (98.91) | 660 (97.49) |  |
| Yes | 47 (1.37) | 30 (1.09) | 17 (2.51) |  |

HR, Hazard Ratio;CI, Confidence Interval.BUN, Blood Urea Nitrogen; RDW,Red Cell Distribution Width；WBC,White Blood Cell count；MCHC, Mean Corpuscular Hemoglobin Concentration;RDW, Red Cell Distribution Width;RBC, Red Blood Cells;MCV, Mean Corpuscular Volume;MCH, Mean Corpuscular Hemoglobin;INR,International Normalized Ratio；PT,Prothrombin Time；PTT,Partial Thromboplastin Time；SBP,Systolic Blood Pressure；DBP,Diastolic Blood Pressure；MBP,Mean Blood Pressure；Resp Rate,Respiratory Rate；SpO₂,Peripheral Oxygen Saturation；AKI,Acute Kidney Injury；CRRT,Continuous Renal Replacement Therapy；SOFA,Sequential Organ Failure Assessment；GCS,Glasgow Coma Scale；SAPSII,Simplified Acute Physiology Score II

Table S3. Univariate cox regression analysis of factors influencing 28-day mortality.

| Variables | *P* | HR (95%CI) |
| --- | --- | --- |
|  |  |  |
| Age | <0.001 | 1.04 (1.03 ~ 1.04) |
| Heart Rate | <0.001 | 1.01 (1.01 ~ 1.02) |
| Resp Rate | <0.001 | 1.05 (1.03 ~ 1.06) |
| SpO_2_ | 0.303 | 0.99 (0.96 ~ 1.01) |
| Glucose | <0.001 | 1.01 (1.01 ~ 1.01) |
| BUN | <0.001 | 1.02 (1.02 ~ 1.02) |
| Potassium | <0.001 | 1.32 (1.19 ~ 1.47) |
| Creatinine | <0.001 | 1.13 (1.08 ~ 1.18) |
| WBC | <0.001 | 1.01 (1.01 ~ 1.02) |
| MCHC | <0.001 | 0.87 (0.83 ~ 0.91) |
| RDW | <0.001 | 1.14 (1.11 ~ 1.17) |
| RBC | <0.001 | 0.80 (0.73 ~ 0.89) |
| MCV | <0.001 | 1.02 (1.01 ~ 1.04) |
| Hemoglobin | <0.001 | 0.93 (0.90 ~ 0.96) |
| Hematocrit | 0.001 | 0.98 (0.97 ~ 0.99) |
| INR | <0.001 | 1.19 (1.11 ~ 1.27) |
| PT | <0.001 | 1.01 (1.01 ~ 1.02) |
| Aniongap | <0.001 | 1.09 (1.07 ~ 1.10) |
| Bicarbonate | <0.001 | 0.93 (0.92 ~ 0.95) |
| Calcium | <0.001 | 0.74 (0.67 ~ 0.81) |
| SOFA | <0.001 | 1.17 (1.12 ~ 1.21) |
| SAPSII | <0.001 | 1.06 (1.05 ~ 1.06) |
| GCS | <0.001 | 0.90 (0.87 ~ 0.92) |
| Charlson Comorbidity Index | <0.001 | 1.19 (1.17 ~ 1.22) |
| Gender |  |  |
| Female |  | 1.00 (Reference) |
| Man | <0.001 | 0.78 (0.67 ~ 0.90) |
| Race |  |  |
| White |  | 1.00 (Reference) |
| Black | 0.827 | 0.97 (0.74 ~ 1.28) |
| Others | <0.001 | 1.64 (1.40 ~ 1.93) |
| Myocardial Infarct |  |  |
| No |  | 1.00 (Reference) |
| Yes | <0.001 | 1.46 (1.20 ~ 1.77) |
| Renal Disease |  |  |
| No |  | 1.00 (Reference) |
| Yes | <0.001 | 1.49 (1.24 ~ 1.78) |
| Liver Disease |  |  |
| No |  | 1.00 (Reference) |
| Yes | 0.018 | 1.46 (1.07 ~ 2.01) |
| Congestive Heart Failure |  |  |
| No |  | 1.00 (Reference) |
| Yes | <0.001 | 1.65 (1.41 ~ 1.94) |
| Dementia |  |  |
| No |  | 1.00 (Reference) |
| Yes | <0.001 | 2.10 (1.61 ~ 2.74) |
| Chronic Pulmonary Disease |  |  |
| No |  | 1.00 (Reference) |
| Yes | 0.019 | 1.25 (1.04 ~ 1.51) |
| Hyperlipidemia |  |  |
| No |  | 1.00 (Reference) |
| Yes | <0.001 | 0.75 (0.65 ~ 0.88) |
| Atrial Fibrillation |  |  |
| No |  | 1.00 (Reference) |
| Yes | <0.001 | 1.74 (1.50 ~ 2.02) |
| Hypertension |  |  |
| No |  | 1.00 (Reference) |
| Yes | <0.001 | 1.40 (1.19 ~ 1.64) |
| AKI |  |  |
| No |  | 1.00 (Reference) |
| Yes | <0.001 | 2.03 (1.65 ~ 2.49) |
| Nitroglycerin |  |  |
| No |  | 1.00 (Reference) |
| Yes | <0.001 | 0.52 (0.38 ~ 0.71) |
| Ventilator |  |  |
| No |  | 1.00 (Reference) |
| Yes | <0.001 | 2.48 (2.05 ~ 2.99) |
| CRRT |  |  |
| No |  | 1.00 (Reference) |
| Yes | 0.005 | 2.00 (1.24 ~ 3.24) |

HR, Hazard Ratio;CI, Confidence Interval.BUN, Blood Urea Nitrogen; RDW,Red Cell Distribution Width；WBC,White Blood Cell count；MCHC, Mean Corpuscular Hemoglobin Concentration;RDW, Red Cell Distribution Width;RBC, Red Blood Cells;MCV, Mean Corpuscular Volume;INR,International Normalized Ratio;PT,Prothrombin Time;Resp Rate,Respiratory Rate;SpO₂,Peripheral Oxygen Saturation;AKI,Acute Kidney Injury；CRRT,Continuous Renal Replacement Therapy；SOFA,Sequential Organ Failure Assessment；GCS,Glasgow Coma Scale；SAPSII,Simplified Acute Physiology Score II

Table S4. Baseline characteristics of patients receiving≤100.3mg day and ＞100.3 mg/day nitroglycerin before and after PSM.

| Variable | Before PSM | | | | |  | After PSM | | | | |
| --- | --- | --- | --- | --- | --- | --- | --- | --- | --- | --- | --- |
|  | Total (n = 320) | ≤100.3mg(n = 145) | ＞100.3mg (n = 175) | *P* | SMD |  | Total (n = 182) | ≤100.3mg(n = 91) | ＞100.3mg (n = 91) | *P* | SMD |
| Age | 71.00 (63.00, 79.25) | 71.00 (63.00, 78.00) | 71.00 (62.50, 80.00) | 0.801 | 0.033 |  | 71.00 (63.00, 80.00) | 73.00 (63.00, 80.00) | 70.00 (62.00, 80.00) | 0.655 | -0.043 |
| Gender, n(%) |  |  |  | 0.017 |  |  |  |  |  | 1.000 |  |
| Female | 129 (40.31) | 48 (33.10) | 81 (46.29) |  | 0.264 |  | 68 (37.36) | 34 (37.36) | 34 (37.36) |  | 0.000 |
| Man | 191 (59.69) | 97 (66.90) | 94 (53.71) |  | -0.264 |  | 114 (62.64) | 57 (62.64) | 57 (62.64) |  | 0.000 |
| Race, n(%) |  |  |  | 0.910 |  |  |  |  |  | 0.948 |  |
| White | 242 (75.62) | 108 (74.48) | 134 (76.57) |  | 0.049 |  | 136 (74.73) | 68 (74.73) | 68 (74.73) |  | 0.000 |
| Black | 23 (7.19) | 11 (7.59) | 12 (6.86) |  | -0.029 |  | 13 (7.14) | 7 (7.69) | 6 (6.59) |  | -0.044 |
| Others | 55 (17.19) | 26 (17.93) | 29 (16.57) |  | -0.037 |  | 33 (18.13) | 16 (17.58) | 17 (18.68) |  | 0.028 |
| **Vital signs** |  |  |  |  |  |  |  |  |  |  |  |
| Heart Rate,bpm | 80.00 (71.00, 88.00) | 80.00 (71.00, 88.00) | 80.00 (70.50, 88.00) | 0.694 | 0.003 |  | 80.00 (70.00, 88.00) | 80.00 (71.00, 87.00) | 79.00 (70.00, 88.00) | 0.509 | -0.038 |
| SBP,mmHg | 120.50 (106.00, 138.00) | 125.00 (108.00, 145.00) | 118.00 (106.00, 134.00) | 0.005 | -0.349 |  | 120.50 (106.00, 138.75) | 121.00 (105.50, 139.00) | 120.00 (106.00, 138.50) | 0.848 | -0.032 |
| DBP,mmHg | 60.00 (52.00, 70.00) | 62.00 (53.00, 70.00) | 60.00 (51.50, 69.00) | 0.401 | -0.151 |  | 60.00 (52.00, 69.75) | 60.00 (52.50, 70.00) | 59.00 (51.50, 69.00) | 0.765 | -0.015 |
| MBP,mmHg | 80.00 (71.00, 91.00) | 83.00 (73.00, 93.00) | 79.00 (71.00, 89.50) | 0.063 | -0.276 |  | 79.00 (71.25, 91.75) | 80.00 (72.50, 92.00) | 79.00 (71.00, 91.00) | 0.792 | -0.033 |
| Resp Rate,bpm | 16.00 (14.00, 18.00) | 16.00 (14.00, 18.00) | 16.00 (14.00, 18.00) | 0.797 | -0.061 |  | 16.00 (14.00, 18.00) | 16.00 (14.00, 18.00) | 16.00 (14.00, 18.00) | 0.727 | -0.036 |
| Temperature,℃ | 36.44 (35.98, 36.83) | 36.50 (36.17, 36.83) | 36.44 (35.91, 36.83) | 0.234 | -0.131 |  | 36.44 (35.90, 36.83) | 36.44 (35.98, 36.81) | 36.44 (35.91, 36.83) | 0.414 | -0.144 |
| SpO2,% | 100.00 (97.00, 100.00) | 100.00 (97.00, 100.00) | 100.00 (98.00, 100.00) | 0.151 | 0.086 |  | 100.00 (97.00, 100.00) | 100.00 (97.00, 100.00) | 100.00 (97.00, 100.00) | 0.658 | -0.083 |
| **Laboratory index** |  |  |  |  |  |  |  |  |  |  |  |
| Glucose,mg/dL | 135.00 (111.00, 173.00) | 134.00 (110.00, 177.00) | 136.00 (114.50, 169.50) | 0.961 | -0.048 |  | 135.50 (114.00, 176.25) | 136.00 (113.00, 178.50) | 135.00 (114.00, 168.00) | 0.660 | -0.040 |
| BUN,mg/dL | 17.00 (13.00, 24.00) | 17.00 (13.00, 23.00) | 18.00 (13.00, 25.00) | 0.374 | -0.041 |  | 17.00 (13.00, 24.00) | 17.00 (13.00, 23.00) | 18.00 (13.00, 24.00) | 0.942 | -0.095 |
| Potassium,mEq/L | 4.20 (3.90, 4.60) | 4.20 (3.90, 4.50) | 4.30 (3.90, 4.65) | 0.029 | 0.250 |  | 4.20 (3.90, 4.50) | 4.20 (3.85, 4.50) | 4.20 (3.90, 4.60) | 0.878 | 0.052 |
| Sodium,mEq/L | 139.00 (136.00, 141.00) | 139.00 (136.00, 141.00) | 139.00 (136.00, 141.00) | 0.807 | 0.118 |  | 139.00 (137.00, 141.00) | 140.00 (136.50, 141.00) | 139.00 (137.00, 141.50) | 0.532 | 0.058 |
| Creatinine,mEq/L | 0.90 (0.70, 1.20) | 0.90 (0.80, 1.20) | 0.90 (0.70, 1.20) | 0.667 | -0.278 |  | 0.90 (0.80, 1.20) | 0.90 (0.80, 1.20) | 1.00 (0.70, 1.20) | 0.760 | -0.104 |
| WBC,K/uL | 10.60 (7.60, 14.30) | 10.10 (7.40, 14.30) | 10.90 (7.75, 14.25) | 0.569 | -0.015 |  | 10.60 (7.75, 14.78) | 9.60 (7.40, 14.25) | 11.20 (8.35, 14.95) | 0.222 | 0.119 |
| MCHC,% | 33.25 (32.20, 34.30) | 33.30 (32.30, 34.50) | 33.20 (32.10, 34.05) | 0.116 | -0.194 |  | 33.10 (32.12, 34.27) | 33.10 (32.15, 34.25) | 33.10 (32.15, 34.25) | 0.559 | -0.105 |
| RDW,% | 13.90 (13.20, 14.83) | 13.90 (13.20, 14.60) | 13.90 (13.20, 14.90) | 0.958 | 0.060 |  | 13.90 (13.20, 15.00) | 13.90 (13.20, 15.00) | 13.90 (13.20, 15.00) | 0.917 | 0.096 |
| RBC,M/uL | 3.27 (2.82, 3.82) | 3.41 (2.86, 3.98) | 3.17 (2.77, 3.75) | 0.030 | -0.239 |  | 3.40 (2.86, 3.90) | 3.40 (2.85, 3.88) | 3.35 (2.88, 3.88) | 0.911 | -0.009 |
| Platelet,K/uL | 166.50 (127.75, 211.00) | 171.00 (143.00, 222.00) | 162.00 (119.00, 203.50) | 0.028 | -0.310 |  | 165.00 (133.25, 218.25) | 162.00 (133.50, 217.50) | 173.00 (133.50, 218.00) | 0.732 | 0.001 |
| MCV,fL | 91.00 (86.00, 95.00) | 91.00 (87.00, 94.00) | 91.00 (86.00, 95.00) | 0.670 | -0.048 |  | 91.00 (87.00, 95.00) | 91.00 (87.50, 95.00) | 91.00 (86.50, 95.50) | 0.735 | -0.031 |
| MCH,pg | 30.10 (28.80, 31.60) | 30.20 (29.20, 31.80) | 30.00 (28.35, 31.50) | 0.090 | -0.160 |  | 30.10 (28.90, 31.78) | 30.20 (29.25, 31.45) | 30.00 (28.30, 31.80) | 0.467 | -0.083 |
| Hematocrit,% | 9.70 (8.50, 11.40) | 10.20 (8.80, 11.80) | 9.30 (8.40, 10.80) | 0.002 | -0.346 |  | 9.85 (8.70, 11.40) | 10.00 (8.80, 11.45) | 9.70 (8.70, 11.25) | 0.647 | -0.055 |
| Hemoglobin,g/dL | 29.60 (25.70, 34.32) | 30.60 (26.70, 35.30) | 28.80 (25.05, 32.80) | 0.007 | -0.290 |  | 30.10 (26.20, 34.80) | 30.30 (26.65, 35.25) | 30.00 (26.00, 34.35) | 0.584 | -0.067 |
| INR | 1.30 (1.10, 1.50) | 1.20 (1.10, 1.50) | 1.30 (1.20, 1.60) | 0.048 | 0.173 |  | 1.30 (1.10, 1.50) | 1.20 (1.10, 1.50) | 1.30 (1.15, 1.55) | 0.367 | 0.156 |
| PT | 14.30 (12.70, 16.62) | 13.90 (12.60, 15.90) | 14.80 (12.95, 17.20) | 0.021 | 0.203 |  | 14.35 (12.70, 16.50) | 14.20 (12.70, 16.10) | 14.80 (12.75, 17.10) | 0.328 | 0.168 |
| PTT | 32.65 (28.70, 40.20) | 32.20 (28.10, 39.60) | 33.20 (29.30, 40.55) | 0.306 | -0.043 |  | 32.75 (28.13, 40.72) | 32.80 (27.60, 40.20) | 32.50 (28.25, 40.70) | 0.864 | 0.089 |
| Chloride,mEq/L | 107.00 (103.00, 111.00) | 107.00 (101.00, 110.00) | 107.00 (104.00, 111.00) | 0.008 | 0.374 |  | 107.00 (103.00, 111.00) | 107.00 (102.00, 111.00) | 107.00 (104.00, 111.00) | 0.648 | 0.142 |
| Aniongap,mEq/L | 12.00 (10.00, 14.00) | 13.00 (10.00, 15.00) | 12.00 (10.00, 14.00) | 0.126 | -0.282 |  | 12.00 (10.00, 14.00) | 12.00 (10.00, 14.00) | 12.00 (10.00, 14.50) | 0.886 | -0.068 |
| Bicarbonate,mEq/L | 23.00 (21.00, 25.00) | 23.00 (22.00, 25.00) | 23.00 (21.00, 25.00) | 0.123 | -0.132 |  | 23.00 (21.00, 25.00) | 23.00 (22.00, 25.00) | 23.00 (21.00, 25.00) | 0.407 | -0.091 |
| Calcium,mg/dL | 8.40 (7.97, 8.90) | 8.50 (8.00, 8.90) | 8.40 (7.95, 8.95) | 0.802 | 0.032 |  | 8.50 (8.00, 8.90) | 8.50 (8.00, 8.90) | 8.40 (7.95, 9.00) | 0.849 | -0.008 |
| **Score** |  |  |  |  |  |  |  |  |  |  |  |
| SOFA | 2.00 (1.00, 4.00) | 2.00 (1.00, 3.00) | 2.00 (1.00, 4.00) | 0.154 | 0.167 |  | 2.00 (1.00, 4.00) | 2.00 (1.00, 4.00) | 2.00 (1.00, 3.00) | 0.502 | -0.147 |
| SAPSII | 37.00 (31.00, 45.00) | 35.00 (30.00, 43.00) | 39.00 (33.00, 47.00) | 0.007 | 0.234 |  | 37.00 (31.25, 45.00) | 37.00 (31.00, 46.50) | 38.00 (32.50, 43.50) | 0.710 | -0.055 |
| GCS | 15.00 (15.00, 15.00) | 15.00 (15.00, 15.00) | 15.00 (15.00, 15.00) | 0.582 | -0.059 |  | 15.00 (15.00, 15.00) | 15.00 (15.00, 15.00) | 15.00 (15.00, 15.00) | 0.848 | 0.020 |
| Charlson Comorbidity Index | 6.00 (4.00, 8.00) | 6.00 (4.00, 8.00) | 6.00 (4.00, 8.00) | 0.335 | 0.101 |  | 6.00 (5.00, 8.00) | 6.00 (5.00, 8.00) | 6.00 (4.00, 8.00) | 0.687 | -0.105 |
| **Complication** |  |  |  |  |  |  |  |  |  |  |  |
| Myocardial Infarct, n (%) |  |  |  | 0.404 |  |  |  |  |  | 0.866 |  |
| No | 241 (75.31) | 106 (73.10) | 135 (77.14) |  | 0.096 |  | 135 (74.18) | 68 (74.73) | 67 (73.63) |  | -0.025 |
| Yes | 79 (24.69) | 39 (26.90) | 40 (22.86) |  | -0.096 |  | 47 (25.82) | 23 (25.27) | 24 (26.37) |  | 0.025 |
| Renal Disease, n (%) |  |  |  | 0.520 |  |  |  |  |  | 0.601 |  |
| No | 244 (76.25) | 113 (77.93) | 131 (74.86) |  | -0.071 |  | 139 (76.37) | 68 (74.73) | 71 (78.02) |  | 0.080 |
| Yes | 76 (23.75) | 32 (22.07) | 44 (25.14) |  | 0.071 |  | 43 (23.63) | 23 (25.27) | 20 (21.98) |  | -0.080 |
| Liver Disease, n (%) |  |  |  | 0.121 |  |  |  |  |  | 1.000 |  |
| No | 309 (96.56) | 137 (94.48) | 172 (98.29) |  | 0.293 |  | 177 (97.25) | 89 (97.80) | 88 (96.70) |  | -0.062 |
| Yes | 11 (3.44) | 8 (5.52) | 3 (1.71) |  | -0.293 |  | 5 (2.75) | 2 (2.20) | 3 (3.30) |  | 0.062 |
| Congestive Heart Failure, n (%) |  |  |  | 0.633 |  |  |  |  |  | 0.873 |  |
| No | 223 (69.69) | 103 (71.03) | 120 (68.57) |  | -0.053 |  | 125 (68.68) | 62 (68.13) | 63 (69.23) |  | 0.024 |
| Yes | 97 (30.31) | 42 (28.97) | 55 (31.43) |  | 0.053 |  | 57 (31.32) | 29 (31.87) | 28 (30.77) |  | -0.024 |
| Peripheral Vascular Disease, n (%) |  |  |  | 0.597 |  |  |  |  |  | 0.628 |  |
| No | 227 (70.94) | 105 (72.41) | 122 (69.71) |  | -0.059 |  | 127 (69.78) | 65 (71.43) | 62 (68.13) |  | -0.071 |
| Yes | 93 (29.06) | 40 (27.59) | 53 (30.29) |  | 0.059 |  | 55 (30.22) | 26 (28.57) | 29 (31.87) |  | 0.071 |
| Dementia, n (%) |  |  |  | 0.801 |  |  |  |  |  | 1.000 |  |
| No | 313 (97.81) | 141 (97.24) | 172 (98.29) |  | 0.080 |  | 177 (97.25) | 88 (96.70) | 89 (97.80) |  | 0.075 |
| Yes | 7 (2.19) | 4 (2.76) | 3 (1.71) |  | -0.080 |  | 5 (2.75) | 3 (3.30) | 2 (2.20) |  | -0.075 |
| Chronic Pulmonary Disease, n (%) |  |  |  | 0.464 |  |  |  |  |  | 1.000 |  |
| No | 238 (74.38) | 105 (72.41) | 133 (76.00) |  | 0.084 |  | 132 (72.53) | 66 (72.53) | 66 (72.53) |  | 0.000 |
| Yes | 82 (25.62) | 40 (27.59) | 42 (24.00) |  | -0.084 |  | 50 (27.47) | 25 (27.47) | 25 (27.47) |  | 0.000 |
| Rheumatic Disease, n (%) |  |  |  | 0.031 |  |  |  |  |  | 0.756 |  |
| No | 303 (94.69) | 133 (91.72) | 170 (97.14) |  | 0.325 |  | 171 (93.96) | 85 (93.41) | 86 (94.51) |  | 0.048 |
| Yes | 17 (5.31) | 12 (8.28) | 5 (2.86) |  | -0.325 |  | 11 (6.04) | 6 (6.59) | 5 (5.49) |  | -0.048 |
| Peptic Ulcer Disease, n (%) |  |  |  | 1.000 |  |  |  |  |  | 1.000 |  |
| No | 316 (98.75) | 143 (98.62) | 173 (98.86) |  | 0.022 |  | 180 (98.9) | 90 (98.90) | 90 (98.90) |  | 0.000 |
| Yes | 4 (1.25) | 2 (1.38) | 2 (1.14) |  | -0.022 |  | 2 (1.1) | 1 (1.10) | 1 (1.10) |  | 0.000 |
| Diabetes, n (%) |  |  |  | 0.161 |  |  |  |  |  | 0.540 |  |
| No | 203 (63.44) | 98 (67.59) | 105 (60.00) |  | -0.155 |  | 114 (62.64) | 55 (60.44) | 59 (64.84) |  | 0.092 |
| Yes | 117 (36.56) | 47 (32.41) | 70 (40.00) |  | 0.155 |  | 68 (37.36) | 36 (39.56) | 32 (35.16) |  | -0.092 |
| Hyperlipidemia, n (%) |  |  |  | 0.331 |  |  |  |  |  | 0.546 |  |
| No | 133 (41.56) | 56 (38.62) | 77 (44.00) |  | 0.108 |  | 74 (40.66) | 35 (38.46) | 39 (42.86) |  | 0.089 |
| Yes | 187 (58.44) | 89 (61.38) | 98 (56.00) |  | -0.108 |  | 108 (59.34) | 56 (61.54) | 52 (57.14) |  | -0.089 |
| Atrial Fibrillation, n (%) |  |  |  | <.001 |  |  |  |  |  | 0.447 |  |
| No | 188 (58.75) | 104 (71.72) | 84 (48.00) |  | -0.475 |  | 111 (60.99) | 58 (63.74) | 53 (58.24) |  | -0.111 |
| Yes | 132 (41.25) | 41 (28.28) | 91 (52.00) |  | 0.475 |  | 71 (39.01) | 33 (36.26) | 38 (41.76) |  | 0.111 |
| Hypertension, n (%) |  |  |  | 0.144 |  |  |  |  |  | 0.656 |  |
| No | 171 (53.44) | 71 (48.97) | 100 (57.14) |  | 0.165 |  | 89 (48.9) | 43 (47.25) | 46 (50.55) |  | 0.066 |
| Yes | 149 (46.56) | 74 (51.03) | 75 (42.86) |  | -0.165 |  | 93 (51.1) | 48 (52.75) | 45 (49.45) |  | -0.066 |
| AKI, n (%) |  |  |  | 0.070 |  |  |  |  |  | 0.672 |  |
| No | 45 (14.06) | 26 (17.93) | 19 (10.86) |  | -0.227 |  | 26 (14.29) | 14 (15.38) | 12 (13.19) |  | -0.065 |
| Yes | 275 (85.94) | 119 (82.07) | 156 (89.14) |  | 0.227 |  | 156 (85.71) | 77 (84.62) | 79 (86.81) |  | 0.065 |
| Treatment |  |  |  |  |  |  |  |  |  |  |  |
| Ventilator, n (%) |  |  |  | <.001 |  |  |  |  |  | 1.000 |  |
| No | 25 (7.81) | 20 (13.79) | 5 (2.86) |  | -0.656 |  | 10 (5.49) | 5 (5.49) | 5 (5.49) |  | 0.000 |
| Yes | 295 (92.19) | 125 (86.21) | 170 (97.14) |  | 0.656 |  | 172 (94.51) | 86 (94.51) | 86 (94.51) |  | 0.000 |
| CRRT, n (%) |  |  |  | 0.356 |  |  |  |  |  | 1.000 |  |
| No | 308 (96.25) | 138 (95.17) | 170 (97.14) |  | 0.118 |  | 175 (96.15) | 87 (95.60) | 88 (96.70) |  | 0.062 |
| Yes | 12 (3.75) | 7 (4.83) | 5 (2.86) |  | -0.118 |  | 7 (3.85) | 4 (4.40) | 3 (3.30) |  | -0.062 |
| **Outcomes** |  |  |  |  |  |  |  |  |  |  |  |
| Hosp Time 28d | 28.00 (28.00, 28.00) | 28.00 (28.00, 28.00) | 28.00 (28.00, 28.00) | 0.309 | 0.203 |  | 28.00 (28.00, 28.00) | 28.00 (28.00, 28.00) | 28.00 (28.00, 28.00) | 0.594 | 0.065 |
| Hosp Outcome 28d, n (%) |  |  |  | 0.349 |  |  |  |  |  | 0.578 |  |
| Survival | 288 (90) | 128 (88.28) | 160 (91.43) |  | 0.113 |  | 168 (92.31) | 83 (91.21) | 85 (93.41) |  | 0.089 |
| Dead | 32 (10) | 17 (11.72) | 15 (8.57) |  | -0.113 |  | 14 (7.69) | 8 (8.79) | 6 (6.59) |  | -0.089 |

HR, Hazard Ratio;CI, Confidence Interval.BUN, Blood Urea Nitrogen; RDW,Red Cell Distribution Width；WBC,White Blood Cell count；MCHC, Mean Corpuscular Hemoglobin Concentration;RDW, Red Cell Distribution Width;RBC, Red Blood Cells;MCV, Mean Corpuscular Volume;MCH, Mean Corpuscular Hemoglobin;INR,International Normalized Ratio；PT,Prothrombin Time；PTT,Partial Thromboplastin Time；SBP,Systolic Blood Pressure；DBP,Diastolic Blood Pressure；MBP,Mean Blood Pressure；Resp Rate,Respiratory Rate；SpO₂,Peripheral Oxygen Saturation；AKI,Acute Kidney Injury；CRRT,Continuous Renal Replacement Therapy；SOFA,Sequential Organ Failure Assessment；GCS,Glasgow Coma Scale；SAPSII,Simplified Acute Physiology Score II
